# Assessment of 16S rRNA gene primers for studying bacterial community structure and function of aging flue-cured tobaccos

**DOI:** 10.1186/s13568-018-0713-1

**Published:** 2018-11-10

**Authors:** Fan Wang, Xiao Men, Ge Zhang, Kaichao Liang, Yuhua Xin, Juan Wang, Aijun Li, Haibo Zhang, Haobao Liu, Lijun Wu

**Affiliations:** 1 CAS Key Laboratory of Biobased Materials, Qingdao Institute of Bioenergy and Bioprocess Technology, Chinese Academy of Sciences, Qingdao, 266101 China; 2 University of Chinese Academy of Sciences, Beijing, 100049 China; 3 Hainan Cigar Research Institute Hainan Provincial Branch of China National Tobacco Corporation, Haikou, 571100 Hainan China; 4 Tobacco Research Institute of Chinese Academy of Agriculture Sciences, Qingdao, 266101 Shandong China; 5 Yunnan Academy of Tobacco Sciences, Kunming, 650106 China

**Keywords:** Microbial community diversity, 16S rRNA gene sequencing, Optimal primer sets, Aging flue-cured tobaccos, PICRUSt, Functional profiling prediction

## Abstract

Selection of optimal primer pairs in 16S rRNA gene sequencing is a pivotal issue in microorganism diversity analysis. However, limited effort has been put into investigation of specific primer sets for analysis of the bacterial diversity of aging flue-cured tobaccos (AFTs), as well as prediction of the function of the bacterial community. In this study, the performance of four primer pairs in determining bacterial community structure based on 16S rRNA gene sequences in AFTs was assessed, and the functions of genes were predicted using Phylogenetic Investigation of Communities by Reconstruction of Unobserved States (PICRUSt). Results revealed that the primer set 799F–1193R covering the amplification region V5V6V7 gave a more accurate picture of the bacterial community structure of AFTs, with lower co-amplification levels of chloroplast and mitochondrial genes, and more genera covered than when using the other primers. In addition, functional gene prediction suggested that the microbiome of AFTs was involved in kinds of interested pathways. A high abundance of functional genes involved in nitrogen metabolism was detected in AFTs, reflecting a high level of bacteria involved in degrading harmful nitrogen compounds and generating nitrogenous nutrients for others. Additionally, the functional genes involved in biosynthesis of valuable metabolites and degradation of toxic compounds provided information that the AFTs possess a huge library of microorganisms and genes that could be applied to further studies. All of these findings provide a significance reference for researchers working on the bacterial diversity assessment of tobacco-related samples.

**Electronic supplementary material:**

The online version of this article (10.1186/s13568-018-0713-1) contains supplementary material, which is available to authorized users.

## Introduction

Microorganisms are dominant drivers of biogeochemical processes (Shinichi et al. 2015), yet a large number of microorganisms (≥ 99%) from the environment remain uncultivated in the laboratory, and these regulate ecosystem processes and even affect our lives (Kaeberlein et al. 2002; Su et al. 2012). Therefore, characterizing microbial community diversity can have theoretical and practical significance in understanding the relationships between microorganism and their hosts, treatment of environmental pollutants, utilization of microbial resources, and human medical health. 16S rRNA gene sequences are considered the predominant and most reliable approach for studying microbial structures in humans, the guts of animals, natural habitats, and the rhizospheres of plants (Beckers et al. 2016). 16S rRNA gene variable regions and the primer sets used for amplification of 16S rRNA gene sequences can result in significantly different bacterial community profiles (Jason et al. 2013; Prosdocimi et al. 2013). Determining optimal primer pairs for 16S rRNA gene sequencing, therefore, plays an important role in microorganism diversity analysis of special hosts.

Several researchers have suggested that the primers V3F and V4R, covering the V3V4 amplification region, should be the preferred primer set for future studies to achieve accurate bacterial community diversities, and a large number of studies have focused on this region (Castelino et al. 2017; Cai et al. 2013; Parulekar et al. 2017; Takahashi et al. 2014). Analogously, for plant samples, not including subterranean plant parts but only above-ground parts such as shoot-tip, leaves, etc., the V3V4 regions has been used predominantly for identifying 16S rRNA gene sequences (Thomas and Sekhar 2017; Tyx et al. 2016; Wang et al. 2018). Nevertheless, due to the homology between bacterial 16S rRNA genes and plant chloroplast and mitochondrial DNA, plant DNA may be coextracted during bacterial DNA extraction from various plant samples, which can cause contaminating sequences (Dyall et al. 2004; Raven 1970). Usually, a high abundance of *Cyanobacteria* at the phylum level, unidentified at the genus level, present in raw sequencing results indicates the occurrence of contaminating sequences from an organellar origin such mitochondrial and/or chloroplast DNA (Beckers et al. 2016). To exclude the interference of contaminating sequences, a common method is to filter out the chloroplast DNA, mitochondrial DNA, and other unknown sequences during data processing. However, this might not reveal the real microbiome structures of samples. To date, although several studies have focused on characterization of bacterial communities in different tobaccos using 16S rRNA gene sequences with the V3V4 and V5V6V7 primers (Huang et al. 2010; Tyx et al. 2016), few report have showed optimized primers for bacterial microbiome investigation of tobaccos, while there are as many as 10,000 chloroplast DNA copies in tobacco leaf cells (Shaver et al. 2006). Microorganisms from aging flue-cured tobaccos (AFTs) would be useful in producing valuable products, treating wastewater, and creating biofuels and a wide range of chemicals and enzymes. There is, therefore, an urgent need to investigate the perfect primers for tobacco bacterial microbiome research.

PICRUSt is an optimal tool to predict the functions of a microbial community’s metagenome from its 16S profile. It predicts genes presented in organisms and uses existing annotations of gene content employing the KEGG (Kyoto Encyclopedia of Genes and Genomes) Orthology and Clusters of Orthologs Groups (Langille et al. 2013). PICRUSt has been used to determine the potential functions of the microbial community in a number of samples (Koo et al. 2017a, b; Wu et al. 2016), which might provide information for development and utilization of microorganism resources. Some studies showed that bacteria in tobacco could generate toxins and pro-inflammatory biomolecules and generate nitrite (Tyx et al. 2016). Thus, characterizing bacterial diversity in tobacco samples and predicting the potential functions of the bacterial community might be a good way to reduce the levels of certain harmful compounds in tobacco and improve the quality of tobacco products, and other products, such as coffee berry, tea, and sauce, etc.

In this study, we used four 16S rRNA gene primer sets to investigate the bacteria in tobacco samples to select the optimal primer sets for characterizing bacterial diversity in tobacco samples. Moreover, the bacterial community function was further predicted using PICRUSt based on 16S rRNA gene sequencing for exploring microorganisms that produced natural products and degraded toxic compounds. The aim of this study was to provide a valuable reference to peer researchers working on bacterial diversity determination of tobacco-related samples, and other plant samples, using 16S rRNA gene sequencing technology, and provide useful information about microorganisms in AFTs for improving the quality and reducing the levels of certain harmful compounds in tobaccos and other fermentation products in further studies.

## Materials and methods

### Experimental design

High-throughput sequencing of 16S rRNA genes was employed to investigate the optimized primer set which could be used to improve the determination of the microbial diversity of AFTs. To evaluate bacterial 16S rRNA gene primer pairs in regard to bacterial diversity coverage and/or accurate taxonomic assignment, four primers pairs (V1F–V3R, V3F–V4R, V4F–V5R, and V5F–V7R) targeting different 16S rRNA gene regions were employed. After individual PCR (Polymerase Chain Reaction) amplification, a total of four fragments were obtained, as shown in Table 1. To minimize non-specific amplification and investigate the potential biases of the Illumina high-throughput sequencing technology, each 16S primer carried a unique eight-base sequence for each sample. Finally, the generated sequencing reads were analyzed by subsequent bioinformatics. In addition, PICRUSt (Langille et al. 2013) was used to predict the functional composition of the metagenome we obtained from the Illumina MiSeq platform analysis of the samples.

**Table 1:** Primers used in current study

Primer pairs	Primer sequence (5′–3′)	M	References
8F	TGGAGAGTTTGATCCTGGCTCAG	V1V2V3	Sun et al. (2014)
533R	TACCGCGGCTGCTGGCAC		
336F	GTACTCCTACGGGAGGCAGCA	V3V4	Munyaka et al. (2015)
806R	GTGGACTACHVGGGTWTCTAAT		
515F	GTGCCAGCMGCCGCGGTAA	V4V5	Tuan et al. (2014)
909R	CCCCGYCAATTCMTTTRAGT		
799F	AACMGGATTAGATACCCKG	V5V6V7	Beckers et al. (2016)
1193R	ACGTCATCCCCACCTTCC		

Primers are indicated as forward (F) or reverse (R)

M, Hypervariable region of the 16S rRNA operon targeted by primer pairs

### Sampling and DNA isolation

Samples were collected from Honghe cigarette factory (Qujing, Yunnan) and Chuxiong cigarette factory (Yuanjiang, Yunnan), respectively. These samples were stored and transported using an ice bath before reaching the laboratory and then stored at − 20 °C. No specific permits were required for this study.

An UltraClean^®^ Soil DNA Isolation Kit (Mobio Inc., Carlsbad, CA, USA) was used to extract total DNA from the collected samples individually according to the manufacturer’s manual. The DNA extracted from three technical replicates of each sample was pooled into one DNA sample to minimize any potential DNA extraction bias. The concentration of each DNA extract was determined with 1% agarose gel electrophoresis and a Thermo NanoDrop 1000 Spectrophotometer (Thermo Scientific, Waltham, MA, USA).

### PCR amplification and Illumina MiSeq sequencing

As been shown in Table 1, the V1V2V3 region of the bacterial 16S rRNA gene was amplified with the universal primers 8F–533R. The PCR program was as follows: 95 °C for 5 min, 26 cycles at 95 °C for 45 s, 55 °C for 50 s, and 72 °C for 45 s, with a final extension of 72 °C for 10 min. The V3V4 region was amplified with the primers 336F–806R following the PCR program 95 °C for 5 min, 27 cycles at 95 °C for 45 s, 50 °C for 50 s, and 72 °C for 45 s, with a final extension of 72 °C for 10 min. The primers 515F–909R covered the V4V5 amplification regions and employed the following PCR program: 95 °C for 5 min, 28 cycles at 95 °C for 45 s, 55 °C for 50 s, and 72 °C for 45 s, with a final extension of 72 °C for 10 min. The V5V6V7 region was amplified with the primers 799F–1193R. The PCR program was 95 °C for 5 min, 27 cycles at 95 °C for 45 s, 55 °C for 50 s, and 72 °C for 45 s, with a final extension of 72 °C for 10 min. All primers contained an 8-nucleotide barcode sequence unique to each sample. All reactions were performed in triplicate in 50 μL volumes containing 5 μL of 10× Pyrobest Buffer, 4 μL of 2.5 mM dNTPs, 2 μL of each primer (10 μM), 0.3 μL of Pyrobest DNA Polymerase (2.5 U/μL, TaKaRa, Japan), and 30 ng of template DNA.

Amplicons were extracted from 2% agarose gels and purified using an AxyPrep DNA Gel Extraction Kit (Axygen Biosciences, Union City, CA, USA) according to the manufacturer’s instructions and quantified using QuantiFluor™-ST (Promega Corporation, Madison, WI, USA). Purified amplicons were pooled in equimolar amounts and paired-end sequenced (2 × 300) on an Illumina MiSeq platform according to standard protocols.

### Bioinformatic analysis

The extraction of high-quality sequences was firstly performed with the QIIME package (Gregory et al. 2010). Raw sequences were selected based on sequence length, quality, primer, and tag, and the low-quality sequences were removed. The Illumina Miseq sequencing data have been deposited in NCBI Sequence Read Archive database with the SRA accession number SRP139912. The unique sequence set was classified into operational taxonomic units (OTUs) under the threshold of 97% identity using UCLUST (Edgar 2010). Chimeric sequences were identified and removed using Usearch (version 8.0.1623). The taxonomy of each 16S rRNA gene sequence was analyzed with UCLUST against the Silva119 16S rRNA gene database using a confidence threshold of 90%.

### Prediction of the functional composition of the metagenome using PICRUSt

The PICRUSt software package (Langille et al. 2013) was used to infer the potential genetic capability and specific contributions of bacterial taxa to the metagenomes of the tobacco samples. PICRUSt requires a phylogenetic tree of marker genes that includes complete reference genomes and the sequences from the samples under study. For this research, we used the KEGG database (Kanehisa 2000) for annotations, and the 16S rRNA gene sequence from each of these genomes was obtained from the United States Department of Energy Joint Genomic Institute’s Integrated Microbial Genomes (IMG) database (Markowitz et al. 2012).

## Results

### Illumina MiSeq sequencing

**Fig. 1:**
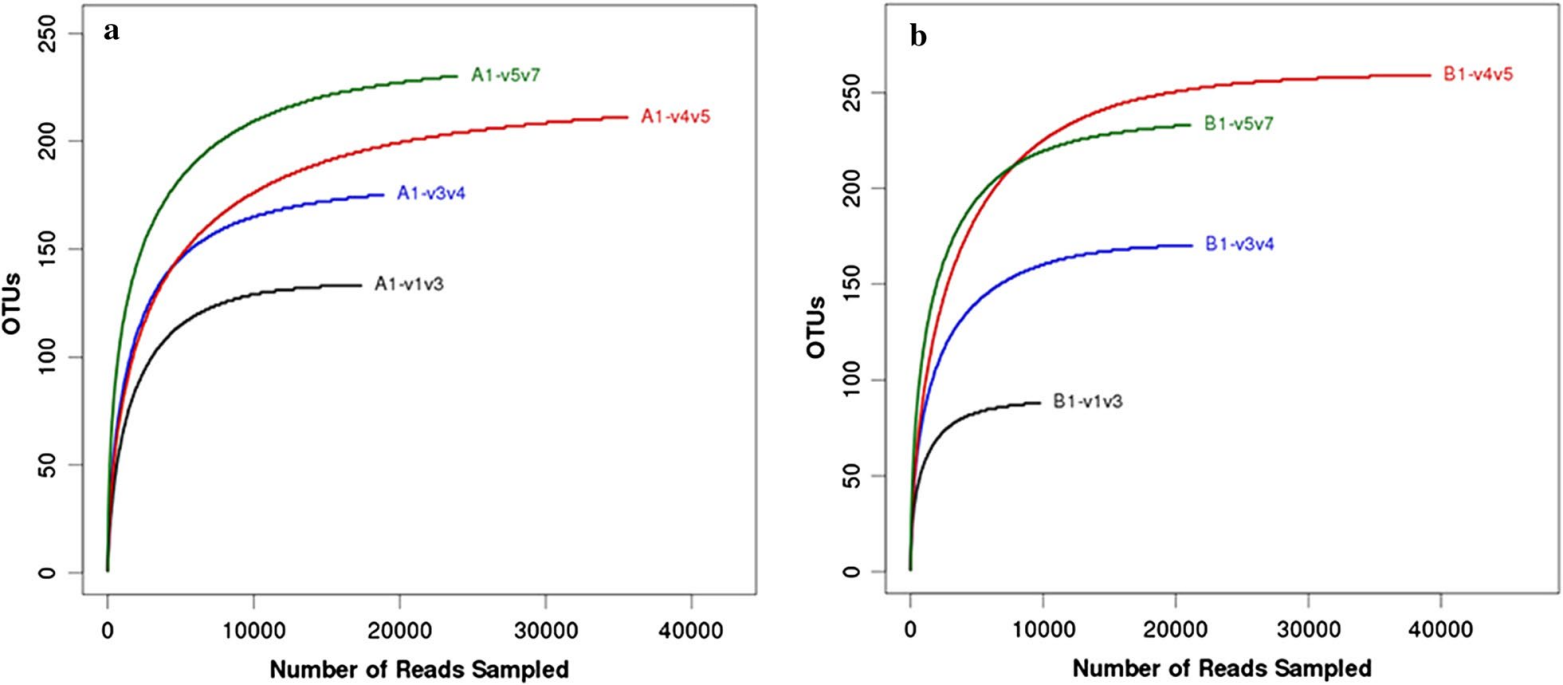
Rarefaction analyses for the observed number of OTUs from the two samples (four regions) at a genetic distance of 3%. Rarefaction curves for each region are displayed in different colors, **a** sample A1 and **b** sample B1

Two samples were collected from Honghe cigarette factory (Qujing, Yunnan) and Chuxiong cigarette factory (Yuanjiang, Yunnan), and sent for high-throughput sequencing of the 16S rRNA gene with four selected bacterial 16S rRNA gene primer pairs (Table 1). Generated reads and OTUs from different primers are shown in Table 2. Rarefaction analysis was performed to evaluate whether the size of the clone library represented the diversity in the original samples (Zhao et al. 2007). In our study, individual rarefaction curves did reflect that the primers give an acceptable measure of species diversity (Fig. 1). The comparison of the primer pairs (four regions) revealed the highest OTU richness and observed species for the V4V5 and V5V6V7 regions. The two samples yielded similar numbers of OTUs with 211 OTUs (A1-V4V5), 259 OTUs (B1-V4V5), 230 OTUs (A1-V5V6V7), and 233 OTUs (B1-V5V6V7), and these were similar to the number of observed species, 209 (A1-V4V5), 258 (B1-V4V5), 226 (A1-V5V6V7), and 232 (B1-V5V6V7), respectively (Table 2).

**Table 2:** Numbers of sequences analyzed for the two samples

Regions	Samples	Reads	OTU	Ace	Chao	Shannon	Simpson	Coverage	Observed species
V1V2V3	A1	17,349	133	133.51	134.99	1.83	0.637187	0.9981	127
	B1	9763	88	89.39	89.52	2.58	0.448405	0.9994	88
V3V4	A1	18,879	175	178.99	179.67	3.21	0.362518	0.9995	173
	B1	21,213	170	171.17	170.37	3.83	0.203652	0.9992	168
V4V5	A1	35,591	211	216.33	214.51	2.64	0.466091	0.9995	209
	B1	39,179	259	259.77	260.03	3.46	0.319225	0.9997	258
V5V6V7	A1	23,913	230	235.58	234.11	5.59	0.038895	0.9989	226
	B1	21,074	233	236.45	238.65	5.41	0.046749	0.9993	232

Operational Taxonomic Units (OTUs), estimated OTU richness, sample coverage, and diversity indices of Shannon and Simpson were calculated from 16S rRNA gene sequences of two samples

### Comparison of diversity and abundance of bacterial taxa

**Fig. 2:**
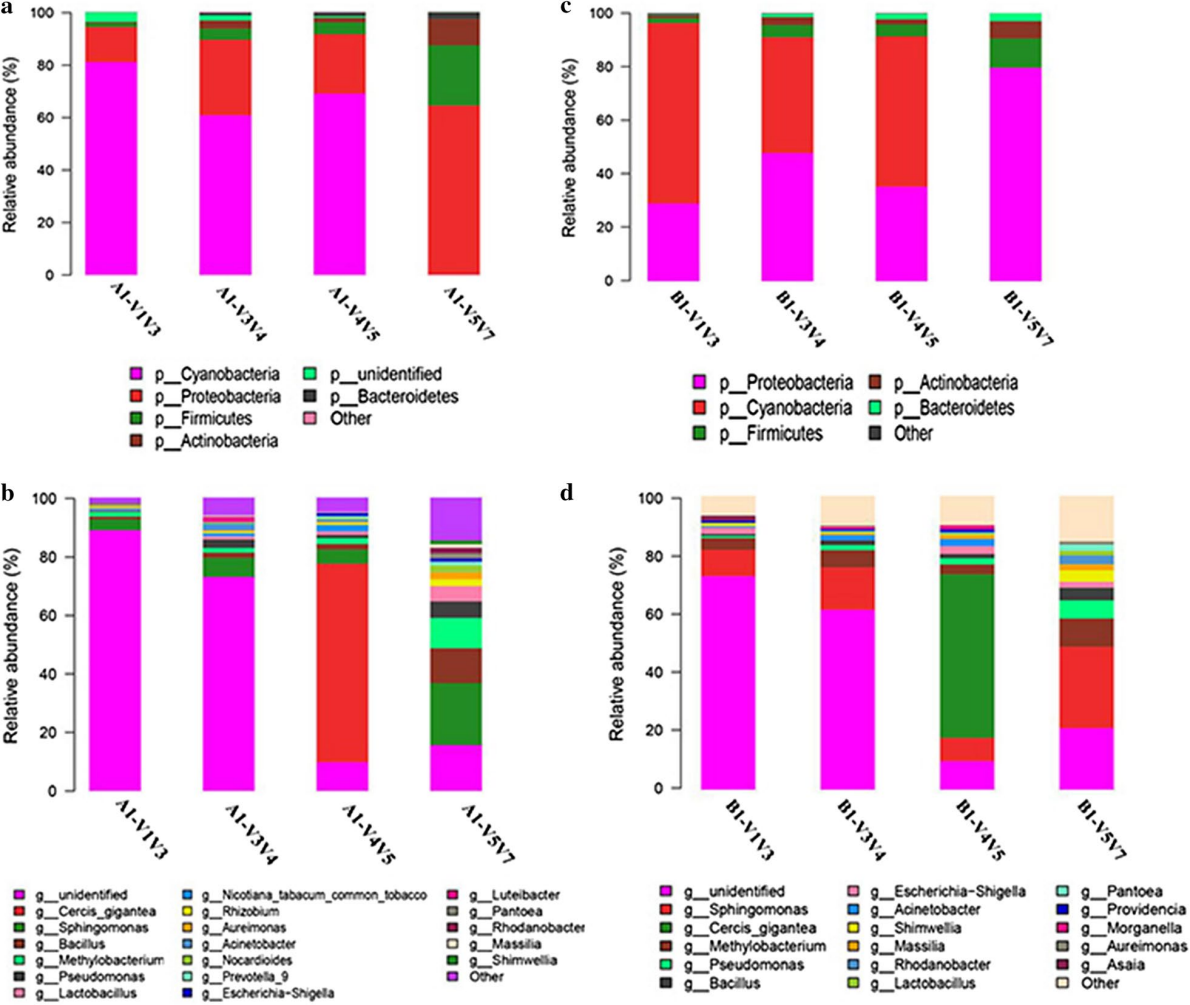
Relative sequence abundance of bacterial phyla (**a**, **c**) and genera (**b**, **d**) associated with different primer sets. Phyla and genera detected as extremely low percentages (< 1%) are not displayed in detail and are summarized as others

**Fig. 3:**
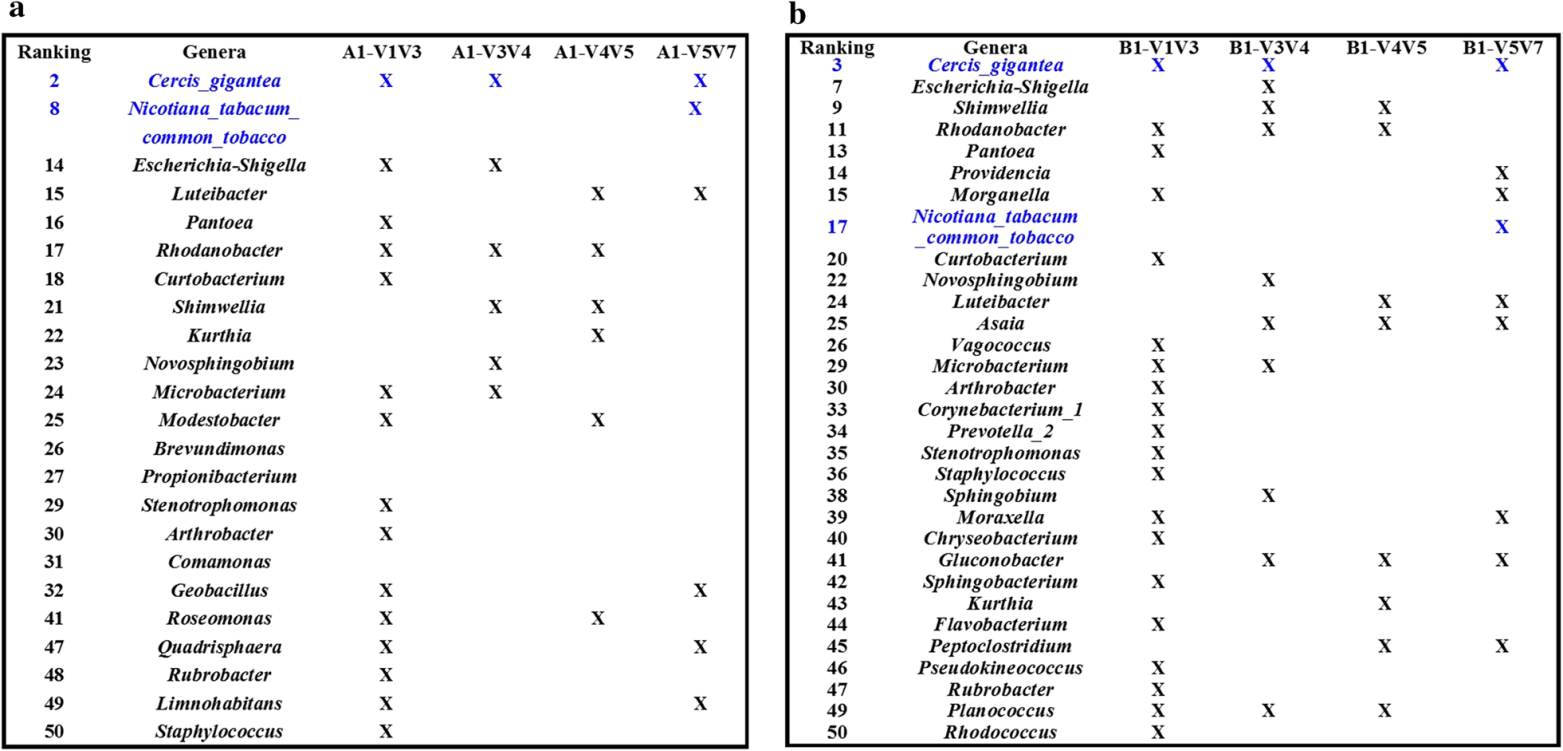
Top 50 genera not detected for each amplification region. The rank was obtained based on the average relative abundance of genera for each amplification region; **a** sample A1 and **b** sample B1; genera not detected are marked with crosses

Samples exhibited a similar range of taxonomic diversity at the phylum and genus levels. Overall, 18 and 16 genera from five phyla were detected with an abundance of at least 0.1% in the two samples, respectively (Fig. 2). At the phylum level (Fig. 2a, b), detection of bacterial taxa was dependent on the choice of 16S regions: *e.g.*, *Cyanobacteria* (V1V2V3 > V3V4 > V4V5 > V5V6V7), *Proteobacteria* and *Actinobacteria* (V5V6V7 > V3V4 > V5V6 > V1V2V3), and *Frimicutes* (V5V6V7 > V3V4or V5V6 > V1V2V3). At genus level also, the results showed that the abundance of detected bacteria was closely related to the covered regions of the selected primer pairs. For example, *Sphingomonas*, *Bacillus*, *Methylobacterium*, *Lactobacillus*, *Nocardioides*, and *Pseudomonas* showed high abundance with primers 799F–1193R, and extremely low abundance with primers 8F–533R. Similar cases were also observed for *Rhizobium*, *Aureimonas*, *Prevotella_9*, and others. *Pantoea* and *Rhodanobacter* displayed high abundance with primers 799F–1193R, but were not detection with the other primers. Unidentified taxa showed high abundance with 8F–533R, but low abundance with primers 515F–909R and 799F–1193R. Of note, *Cercis gigantea* and *Nicotiana tabacum* (common tobacco) were two plant species detected. *Cercis gigantea* was detected only with primers 515F–909R with high abundance, and *N. tabacum* (common tobacco) was not detected with primers 799F–1193R. Figure 3 further illustrates that primers 799F–1193R amplified more genera than the other primers from the top 50 genera.

### Primers for the V5–V7 region are highly recommended

The primers 799F–1193R covering region V5V6V7 performed better than the others, which was evidenced as follows. First, primers 799F–1193R had low co-amplification levels of chloroplast and mitochondrial genes, which was concluded from the absence of detection of *Cyanobacteria* at the phylum level and *C. gigantea* and *N. tabacum* (common tobacco) at the genus level (Fig. 2). Second, primers 799F–1193R covered more genera than the other primers (Figs. 2c, d, 3). Third, primers 799F–1193R gave the highest OTU richness (Table 2). To achieve a relatively accurate assessment of bacterial community structure and diversity, here we highly recommend 799F–1193R as the preferred primers for future studies of tobacco-related samples, and even other plant samples, such as ensilage, etc.

### Functional gene prediction

**Fig. 4:**
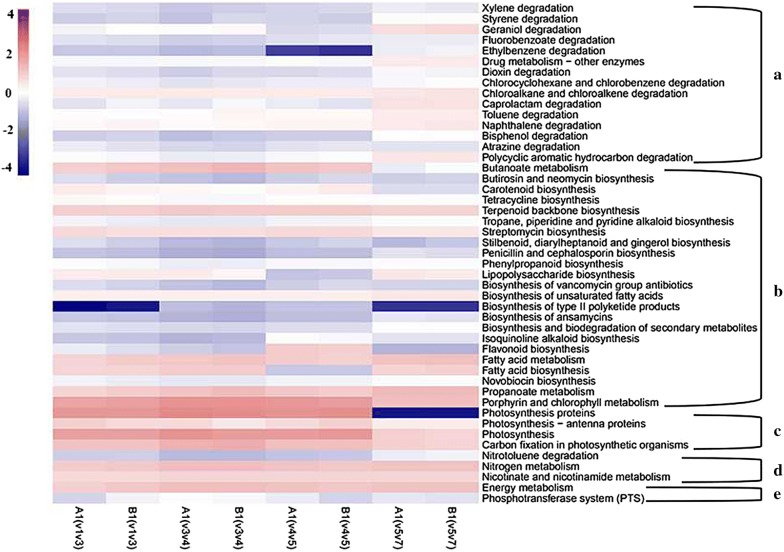
Heatmap of predicted functional pathways assigned to interesting genes investigated in bacteria of AFTs. Functional genes are related to degradation of harmful compounds (**a**), biosynthesis of valuable metabolites (**b**), photosynthesis (**c**), nitrogen metabolism (**d**), and energy metabolism (**e**)

AFTs microbiome functions were predicted with PICRUSt and annotated using the KEGG database, which showed that the microbiome of AFTs is involved in diverse pathways at level 3 KEGG Orthology (Additional file 1: Tables S1–S4). Functional genes related to degradation of harmful compounds, biosynthesis of valuable metabolites, photosynthesis, nitrogen metabolism, and energy metabolism were interestingly investigated. The heatmap obtained from the analysis of interesting functional genes is presented in Fig. 4. The abundance of photosynthesis-related genes detected, especially photosynthesis proteins, was much lower using the V5V6V7 region, and other genes showed slightly significant differences in abundance when using different 16S regions. This analysis demonstrated that primers 799F–1193R covering the V5V6V7 region as the preferred sequencing region should be used in future tobacco-related samples studies. Moreover, several remarkable genes were involved in biosynthesis of flavors and fragrances, and degradation of harmful compounds, especially nicotine, which could be employed to enhance the quality of tobacco leaves during the aging process. Besides, the results concluded that bacteria on AFTs are important microorganisms and natural resources of genes for future exploration.

## Discussion

There is no uniform argument for which primer set we should employ for particular samples. Studies have suggested that the V3, V4, or V3V4 regions are highly recommended for work employing 16S rRNA gene sequences as these provided adequate and accurate information for taxonomic classification of bacterial communities (Castelino et al. 2017; Cai et al. 2013). The V3V4 regions are also highly recommended by genome sequencing companies. However, it is confusing to find that similar type of samples sometimes use different primer sets (Huang et al. 2010; Tyx et al. 2016), while different types of samples use similar primers (Huang et al. 2010; Thomas and Sekhar 2017). For dealing with the problem of selection of preferred primer sets in the characterization of bacterial diversity of AFTs by 16S rRNA gene sequence, we employed four primer sets in this study. Conclusively, the choice of primer set could have a dramatic impact on the resulting accuracy of bacterial community structure determination. The results showed that the primers 799F-1193R had low co-amplification levels of chloroplast and mitochondrial genes, and also covered more genera than other primers. Moreover, the conclusion was also confirmed by the results of functional gene prediction. It is obvious that the abundance of functional genes related to photosynthesis was far lower using the V5V6V7 region than the other 16S regions. Therefore, we highly recommend that the preferred primers 799F–1193R are used to study the accurate diversity of tobacco-related samples, and even other plant samples in future studies, and the plant samples are not only the above ground parts of plants, but also the subterranean parts, as suggested by several other reports (Beckers et al. 2016; Huang et al. 2010; Sun et al. 2008).

A large number of reports have highlighted the importance of microbial communities in degradation of harmful compounds, natural product biosynthesis, and other metabolism processes (Abia et al. 2018; Chen et al. 2016; Thelusmond et al. 2016; Wang et al. 2016; Yan et al. 2015). PICRUSt provided a convenient way to predict the functional composition of a metagenome using a database of reference genomes. In addition, it gave useful insights into the large number of uncultured microorganisms (Langille et al. 2013). In the present study, we employed PICRUSt to predict the functional gene contents and abundances among microbial communities on AFTs, and revealed several attractive genes relevant to (1) photosynthesis, (2) nitrogen metabolism, (3) biosynthesis of valuable metabolites, (4) degradation of toxic compounds, and (5) energy metabolism. Genes relevant to photosynthesis are possessed by plants, algae, and cyanobacteria, and were investigated in this paper, leading to the recommendation of primers 799F–1193R as the preferred sequencing region in future tobacco-related samples. Moreover, in other reports, genes relevant to photosynthesis were candidate genes involved in cold responses, energy metabolism, and carbohydrate metabolism (Bryant and Frigaard 2006; Legrand et al. 2013). Genes relevant to nitrogen metabolism are pivotal in the tobacco-related samples. It is well known that nicotine and tobacco-specific nitrosamines, the major nitrogen compounds in tobacco plants, play a critical role in smoking addiction and are harmful to humans (Liu et al. 2015; Wei et al. 2014). PICRUSt offers a promising way to discover harmful nitrogen compound-degrading microorganisms for improving the quality of tobaccos, treating tobacco waste, and producing valuable intermediates of these compounds. Genes relevant to biosynthesis of valuable metabolites, especially aroma compounds, would have a bright prospect when exploring microorganisms that produce natural products. Similarly, genes relevant to degradation of toxic compounds, which include polycyclic aromatic hydrocarbons and other pollutants, have the potential to deal with contaminants of AFTs. Genes relevant to energy metabolism, which is a process of generating energy for the whole the organism, have a key role in the above actions of bacteria on AFTs. This might justify the conclusion that AFTs are a treasury of microbes that could be widely used in all walks of life.

In this study, the performance of four primer pairs for determination of bacterial community structure on AFTs using 16S rRNA gene sequences were evaluated experimentally, and the corresponding functional genes were predicted using PICRUSt. Results suggested that the amplification region V5V6V7 with primers 799F–1193R generated a more accurate picture of the bacterial community structure on AFTs. First, primers 799F–1193R had low co-amplification levels of chloroplast and mitochondrial genes, which was concluded from the result of low abundance of *Cyanobacteria* at the phylum level and no *C. gigantea* or *N. tabacum* (common tobacco) at the genus level. Then, primers 799F–1193R covered more genera than the other primers. Finally, the PICRUSt results displayed that the abundance of photosynthesis-related genes was much lower in the amplification region V5V6V7. Therefore, we highly recommend that primers 799F–1193R should be used in future studies of plant samples, especially tobacco-related samples. In addition, functional gene prediction showed that the microbiome on AFTs was involved in a number of pathways. The study paid most attention to functional genes related to photosynthesis, nitrogen metabolism, biosynthesis of valuable metabolites, degradation of toxic compounds, and energy metabolism. A high abundance of functional genes involved in nitrogen metabolism was observed, reflecting a high level of bacteria on AFTs involved in degrading harmful nitrogen compounds and generating nitrogenous nutrients for others. Additionally, the functional genes involved in biosynthesis of valuable metabolites and degradation of toxic compounds provided information that the AFTs possess a huge library of microorganisms and genes that could be applied to further studies.

## Additional file

**Additional file 1.** Additional tables.

## Abbreviations

AFTs: aging flue-cured tobaccos
PICRUSt: Phylogenetic Investigation of Communities by Reconstruction of Unobserved States
KEGG: Kyoto Encyclopedia of Genes and Genomes
PCR: Polymerase Chain Reaction
IMG: Integrated Microbial Genomes
