# Supplementary material for: Assessment of 16S rRNA gene primers for studying bacterial community structure and function of aging flue-cured tobaccos
Source: AMB Express. 2018 Nov 10;8:182. doi: 10.1186/s13568-018-0713-1 (PMC6230335; doi:10.1186/s13568-018-0713-1)
Supplement: Supplementary file 1 — Additional file 1. Additional tables. [file 13568_2018_713_MOESM1_ESM.pdf]

AMB Express

Additional file 1 for

Assessment of 16S rRNA gene primers for studying bacterial community structure and function of aging flue-cured tobaccos

Fan Wang[1,2], Xiao Men[1,2], Ge Zhang[1,2], Kaichao Liang[3], Yuhua Xin[3], Juan Wang[3], Aijun Li[3], Haibo Zhang[1,2*], Haobao Liu[3,4*], Lijun Wu[5*]

[1]CAS Key Laboratory of Biobased Materials, Qingdao Institute of Bioenergy and Bioprocess Technology, Chinese Academy of Sciences, Qingdao 266101, China

[2]University of Chinese Academy of Sciences, Beijing 100049, China

[3] Hainan Cigar Research Institute Hainan Provincial Branch of China National Tobacco Corporation, Haikou 571100, Hainan, China

[4] Tobacco Research Institute of Chinese Academy of Agriculture Sciences, Qingdao 266101, Shandong, China

[5]Yunnan Academy of Tobacco Sciences, Kunming 650106, China;

* Corresponding author

E-mail: zhanghb@qibebt.ac.cn (H. Zhang), liuhaobao@caas.cn (H. Liu), 2818429250@qq.com (L. Wu)

Tel: +86 139 6978 0438 (H. Zhang), +86 182 5325 0799 (H. Liu), +86 136 5883 4314 (L. Wu)

Fax: +86 0532 8066 2765 (H. Zhang), +86 0532 8066 2766 (H. Liu), +86 0871 6586 9642 (L. Wu)

Table Captions

Table S1 Results of PICRUSt at level 3 KEGG Orthology using the V1-V3 amplification regions.

Table S2 Results of PICRUSt at level 3 KEGG Orthology using the V3-V4 amplification regions.

Table S3 Results of PICRUSt at level 3 KEGG Orthology using the V4-V5 amplification regions.

Table S4 Results of PICRUSt at level 3 KEGG Orthology using the V5-V7 amplification regions.

Table S1 Results of PICRUSt at level 3 KEGG Orthology using the V1-V3 amplification regions.

OTU ID	B1(V1V3)	A1(V1V3)	KEGG Pathways
1,1,1-Trichloro-2,2-bis(4-chlorophenyl) ethane (DDT) degradation	17	19	Metabolism; Xenobiotics Biodegradation and Metabolism; 1,1,1-Trichloro-2,2-bis(4-chlorophenyl) ethane (DDT) degradation
ABC transporters	465709	811389	Environmental Information Processing; Membrane Transport; ABC transporters
Adipocytokine signaling pathway	9552	15886	Organismal Systems; Endocrine System; Adipocytokine signaling pathway
African trypanosomiasis	1359	1359	Human Diseases; Infectious Diseases; African trypanosomiasis
Alanine, aspartate and glutamate metabolism	114209	196658	Metabolism; Amino Acid Metabolism; Alanine, aspartate and glutamate metabolism
Aldosterone-regulated sodium reabsorption	0	2	Organismal Systems; Excretory System; Aldosterone-regulated sodium reabsorption
Alzheimer's disease	10020	13589	Human Diseases; Neurodegenerative Diseases; Alzheimer's disease
Amino acid metabolism	37618	68098	Unclassified; Metabolism; Amino acid metabolism
Amino acid related enzymes	160923	273469	Metabolism; Amino Acid Metabolism; Amino acid related enzymes
Amino sugar and nucleotide sugar metabolism	148916	261893	Metabolism; Carbohydrate Metabolism; Amino sugar and nucleotide sugar metabolism
Aminoacyl-tRNA biosynthesis	137222	238905	Genetic Information Processing; Translation; Aminoacyl-tRNA biosynthesis
Aminobenzoate degradation	36562	53925	Metabolism; Xenobiotics Biodegradation and Metabolism; Aminobenzoate degradation
Amoebiasis	3773	7181	Human Diseases; Infectious Diseases; Amoebiasis
Amyotrophic lateral sclerosis (ALS)	3612	3013	Human Diseases; Neurodegenerative Diseases; Amyotrophic lateral sclerosis (ALS)
Antigen processing and presentation	4179	7862	Organismal Systems; Immune System; Antigen processing and presentation
Apoptosis	2009	1784	Cellular Processes; Cell Growth and Death; Apoptosis
Arachidonic acid metabolism	10041	16193	Metabolism; Lipid Metabolism; Arachidonic acid metabolism
Arginine and proline metabolism	170133	297529	Metabolism; Amino Acid Metabolism; Arginine and proline metabolism
Ascorbate and aldarate metabolism	18444	27405	Metabolism; Carbohydrate Metabolism; Ascorbate and aldarate metabolism
Atrazine degradation	11917	22602	Metabolism; Xenobiotics Biodegradation and Metabolism; Atrazine degradation
Bacterial chemotaxis	37441	41220	Cellular Processes; Cell Motility; Bacterial chemotaxis
Bacterial invasion of epithelial cells	3343	6882	Human Diseases; Infectious Diseases; Bacterial invasion of epithelial cells
Bacterial motility proteins	174772	275385	Cellular Processes; Cell Motility; Bacterial motility proteins
Bacterial secretion system	84775	128020	Environmental Information Processing; Membrane Transport; Bacterial secretion system
Bacterial toxins	9491	15692	Environmental Information Processing; Signaling Molecules and Interaction; Bacterial toxins
Basal transcription factors	137	99	Genetic Information Processing; Transcription; Basal transcription factors
Base excision repair	50743	84223	Genetic Information Processing; Replication and Repair; Base excision repair
Benzoate degradation	37603	45891	Metabolism; Xenobiotics Biodegradation and Metabolism; Benzoate degradation
Betalain biosynthesis	43	20	Metabolism; Biosynthesis of Other Secondary Metabolites; Betalain biosynthesis
Bile secretion	77	87	Organismal Systems; Digestive System; Bile secretion
Biosynthesis and biodegradation of secondary metabolites	10625	17044	Unclassified; Metabolism; Biosynthesis and biodegradation of secondary metabolites
Biosynthesis of 12-, 14- and 16-membered macrolides	3	2	Metabolism; Metabolism of Terpenoids and Polyketides; Biosynthesis of 12-, 14- and 16-membered macrolides
Biosynthesis of ansamycins	5630	8841	Metabolism; Metabolism of Terpenoids and Polyketides; Biosynthesis of ansamycins
Biosynthesis of siderophore group nonribosomal peptides	5855	8959	Metabolism; Metabolism of Terpenoids and Polyketides; Biosynthesis of siderophore group nonribosomal peptides

Biosynthesis of type II polyketide backbone	3	6	Metabolism; Metabolism of Terpenoids and Polyketides; Biosynthesis of type II polyketide backbone
Biosynthesis of type II polyketide products	134	64	Metabolism; Metabolism of Terpenoids and Polyketides; Biosynthesis of type II polyketide products
Biosynthesis of unsaturated fatty acids	30282	50104	Metabolism; Lipid Metabolism; Biosynthesis of unsaturated fatty acids
Biosynthesis of vancomycin group antibiotics	8020	14871	Metabolism; Metabolism of Terpenoids and Polyketides; Biosynthesis of vancomycin group antibiotics
Biotin metabolism	21945	38598	Metabolism; Metabolism of Cofactors and Vitamins; Biotin metabolism
Bisphenol degradation	8402	10383	Metabolism; Xenobiotics Biodegradation and Metabolism; Bisphenol degradation
Bladder cancer	477	401	Human Diseases; Cancers; Bladder cancer
Butanoate metabolism	86073	119029	Metabolism; Carbohydrate Metabolism; Butanoate metabolism
Butirosin and neomycin biosynthesis	7640	14573	Metabolism; Biosynthesis of Other Secondary Metabolites; Butirosin and neomycin biosynthesis
C5-Branched dibasic acid metabolism	35149	57608	Metabolism; Carbohydrate Metabolism; C5-Branched dibasic acid metabolism
Caffeine metabolism	84	105	Metabolism; Biosynthesis of Other Secondary Metabolites; Caffeine metabolism
Calcium signaling pathway	3216	6850	Environmental Information Processing; Signal Transduction; Calcium signaling pathway
Caprolactam degradation	15394	16128	Metabolism; Xenobiotics Biodegradation and Metabolism; Caprolactam degradation
Carbohydrate digestion and absorption	3643	7194	Organismal Systems; Digestive System; Carbohydrate digestion and absorption
Carbohydrate metabolism	31712	59603	Unclassified; Metabolism; Carbohydrate metabolism
Carbon fixation in photosynthetic organisms	91869	168548	Metabolism; Energy Metabolism; Carbon fixation in photosynthetic organisms
Carbon fixation pathways in prokaryotes	109245	173979	Metabolism; Energy Metabolism; Carbon fixation pathways in prokaryotes
Cardiac muscle contraction	3013	3247	Organismal Systems; Circulatory System; Cardiac muscle contraction
Carotenoid biosynthesis	28018	56352	Metabolism; Metabolism of Terpenoids and Polyketides; Carotenoid biosynthesis
Cell cycle - Caulobacter	74323	126329	Cellular Processes; Cell Growth and Death; Cell cycle - Caulobacter
Cell division	9961	16585	Unclassified; Cellular Processes and Signaling; Cell division
Cell motility and secretion	36694	63998	Unclassified; Cellular Processes and Signaling; Cell motility and secretion
Cellular antigens	9640	15925	Environmental Information Processing; Signaling Molecules and Interaction; Cellular antigens
Chagas disease (American trypanosomiasis)	1323	1300	Human Diseases; Infectious Diseases; Chagas disease (American trypanosomiasis)
Chaperones and folding catalysts	147552	261322	Genetic Information Processing; Folding, Sorting and Degradation; Chaperones and folding catalysts
Chloroalkane and chloroalkene degradation	35209	56704	Metabolism; Xenobiotics Biodegradation and Metabolism; Chloroalkane and chloroalkene degradation
Chlorocyclohexane and chlorobenzene degradation	14023	23928	Metabolism; Xenobiotics Biodegradation and Metabolism; Chlorocyclohexane and chlorobenzene degradation
Chromosome	200954	354658	Genetic Information Processing; Replication and Repair; Chromosome
Circadian rhythm - plant	409	269	Organismal Systems; Environmental Adaptation; Circadian rhythm - plant
Citrate cycle (TCA cycle)	71726	108043	Metabolism; Carbohydrate Metabolism; Citrate cycle (TCA cycle)
Clavulanic acid biosynthesis	2	30	Metabolism; Biosynthesis of Other Secondary Metabolites; Clavulanic acid biosynthesis
Colorectal cancer	1865	1736	Human Diseases; Cancers; Colorectal cancer
Cyanoamino acid metabolism	32204	55319	Metabolism; Metabolism of Other Amino Acids; Cyanoamino acid metabolism
Cysteine and methionine metabolism	100551	168544	Metabolism; Amino Acid Metabolism; Cysteine and methionine metabolism
Cytochrome P450	55	36	Metabolism; Enzyme Families; Cytochrome P450
Cytoskeleton proteins	45187	83590	Cellular Processes; Cell Motility; Cytoskeleton proteins

D-Alanine metabolism	9790	16110	Metabolism; Metabolism of Other Amino Acids; D-Alanine metabolism
D-Arginine and D-ornithine metabolism	257	251	Metabolism; Metabolism of Other Amino Acids; D-Arginine and D-ornithine metabolism
D-Glutamine and D-glutamate metabolism	18242	31753	Metabolism; Metabolism of Other Amino Acids; D-Glutamine and D-glutamate metabolism
DNA repair and recombination proteins	337127	584365	Genetic Information Processing; Replication and Repair; DNA repair and recombination proteins
DNA replication	65272	110366	Genetic Information Processing; Replication and Repair; DNA replication
DNA replication proteins	121451	208062	Genetic Information Processing; Replication and Repair; DNA replication proteins
Dioxin degradation	9772	16109	Metabolism; Xenobiotics Biodegradation and Metabolism; Dioxin degradation
Drug metabolism - cytochrome P450	38076	64063	Metabolism; Xenobiotics Biodegradation and Metabolism; Drug metabolism - cytochrome P450
Drug metabolism - other enzymes	17929	26626	Metabolism; Xenobiotics Biodegradation and Metabolism; Drug metabolism - other enzymes
Electron transfer carriers	5085	8317	Unclassified; Cellular Processes and Signaling; Electron transfer carriers
Endocytosis	10	12	Cellular Processes; Transport and Catabolism; Endocytosis
Energy metabolism	82460	129208	Unclassified; Metabolism; Energy metabolism
Epithelial cell signaling in Helicobacter pylori infection	7872	14792	Human Diseases; Infectious Diseases; Epithelial cell signaling in Helicobacter pylori infection
Ether lipid metabolism	526	311	Metabolism; Lipid Metabolism; Ether lipid metabolism
Ethylbenzene degradation	6503	9329	Metabolism; Xenobiotics Biodegradation and Metabolism; Ethylbenzene degradation
Fatty acid biosynthesis	63434	107108	Metabolism; Lipid Metabolism; Fatty acid biosynthesis
Fatty acid elongation in mitochondria	1	0	Metabolism; Lipid Metabolism; Fatty acid elongation in mitochondria
Fatty acid metabolism	71833	107393	Metabolism; Lipid Metabolism; Fatty acid metabolism
Fc gamma R-mediated phagocytosis	10	12	Organismal Systems; Immune System; Fc gamma R-mediated phagocytosis
Flagellar assembly	30243	21357	Cellular Processes; Cell Motility; Flagellar assembly
Flavone and flavonol biosynthesis	53	11	Metabolism; Biosynthesis of Other Secondary Metabolites; Flavone and flavonol biosynthesis
Flavonoid biosynthesis	10253	20953	Metabolism; Biosynthesis of Other Secondary Metabolites; Flavonoid biosynthesis
Fluorobenzoate degradation	9578	16019	Metabolism; Xenobiotics Biodegradation and Metabolism; Fluorobenzoate degradation
Folate biosynthesis	61916	108625	Metabolism; Metabolism of Cofactors and Vitamins; Folate biosynthesis
Fructose and mannose metabolism	93259	161732	Metabolism; Carbohydrate Metabolism; Fructose and mannose metabolism
Function unknown	199274	324384	Unclassified; Poorly Characterized; Function unknown
G protein-coupled receptors	7	1	Environmental Information Processing; Signaling Molecules and Interaction; G protein-coupled receptors
Galactose metabolism	44329	72912	Metabolism; Carbohydrate Metabolism; Galactose metabolism
General function prediction only	505671	883436	Unclassified; Poorly Characterized; General function prediction only
Geraniol degradation	22029	25716	Metabolism; Metabolism of Terpenoids and Polyketides; Geraniol degradation
Germination	277	144	Unclassified; Cellular Processes and Signaling; Germination
Glutamatergic synapse	12978	23452	Organismal Systems; Nervous System; Glutamatergic synapse
Glutathione metabolism	73121	121663	Metabolism; Metabolism of Other Amino Acids; Glutathione metabolism
Glycan biosynthesis and metabolism	9480	15955	Unclassified; Metabolism; Glycan biosynthesis and metabolism
Glycerolipid metabolism	44801	78141	Metabolism; Lipid Metabolism; Glycerolipid metabolism
Glycerophospholipid metabolism	58555	94606	Metabolism; Lipid Metabolism; Glycerophospholipid metabolism
Glycine, serine and threonine metabolism	94965	154230	Metabolism; Amino Acid Metabolism; Glycine, serine and threonine metabolism
Glycolysis / Gluconeogenesis	148426	259407	Metabolism; Carbohydrate Metabolism; Glycolysis /

			Gluconeogenesis
Glycosaminoglycan degradation	1342	881	Metabolism; Glycan Biosynthesis and Metabolism; Glycosaminoglycan degradation
Glycosphingolipid biosynthesis - ganglio series	827	572	Metabolism; Glycan Biosynthesis and Metabolism; Glycosphingolipid biosynthesis - ganglio series
Glycosphingolipid biosynthesis - globo series	1450	1071	Metabolism; Glycan Biosynthesis and Metabolism; Glycosphingolipid biosynthesis - globo series
Glycosphingolipid biosynthesis - lacto and neolacto series	24	9	Metabolism; Glycan Biosynthesis and Metabolism; Glycosphingolipid biosynthesis - lacto and neolacto series
Glycosyltransferases	75311	140843	Metabolism; Glycan Biosynthesis and Metabolism; Glycosyltransferases
Glyoxylate and dicarboxylate metabolism	79617	123168	Metabolism; Carbohydrate Metabolism; Glyoxylate and dicarboxylate metabolism
GnRH signaling pathway	10	12	Organismal Systems; Endocrine System; GnRH signaling pathway
Hematopoietic cell lineage	0	4	Organismal Systems; Immune System; Hematopoietic cell lineage
Histidine metabolism	64458	110144	Metabolism; Amino Acid Metabolism; Histidine metabolism
Homologous recombination	99218	172296	Genetic Information Processing; Replication and Repair; Homologous recombination
Huntington's disease	13897	20828	Human Diseases; Neurodegenerative Diseases; Huntington's disease
Hypertrophic cardiomyopathy (HCM)	70	41	Human Diseases; Cardiovascular Diseases; Hypertrophic cardiomyopathy (HCM)
Indole alkaloid biosynthesis	37	19	Metabolism; Biosynthesis of Other Secondary Metabolites; Indole alkaloid biosynthesis
Influenza A	1865	1737	Human Diseases; Infectious Diseases; Influenza A
Inorganic ion transport and metabolism	29100	39717	Unclassified; Cellular Processes and Signaling; Inorganic ion transport and metabolism
Inositol phosphate metabolism	18230	27960	Metabolism; Carbohydrate Metabolism; Inositol phosphate metabolism
Insulin signaling pathway	20960	42419	Organismal Systems; Endocrine System; Insulin signaling pathway
Ion channels	4335	7691	Environmental Information Processing; Signaling Molecules and Interaction; Ion channels
Isoflavonoid biosynthesis	6	2	Metabolism; Biosynthesis of Other Secondary Metabolites; Isoflavonoid biosynthesis
Isoquinoline alkaloid biosynthesis	5829	9147	Metabolism; Biosynthesis of Other Secondary Metabolites; Isoquinoline alkaloid biosynthesis
Leishmaniasis	1	0	Human Diseases; Infectious Diseases; Leishmaniasis
Limonene and pinene degradation	25098	32657	Metabolism; Metabolism of Terpenoids and Polyketides; Limonene and pinene degradation
Linoleic acid metabolism	3443	1997	Metabolism; Lipid Metabolism; Linoleic acid metabolism
Lipid biosynthesis proteins	92518	155749	Metabolism; Lipid Metabolism; Lipid biosynthesis proteins
Lipid metabolism	22002	39518	Unclassified; Metabolism; Lipid metabolism
Lipoic acid metabolism	16093	30382	Metabolism; Metabolism of Cofactors and Vitamins; Lipoic acid metabolism
Lipopolysaccharide biosynthesis	32901	55010	Metabolism; Glycan Biosynthesis and Metabolism; Lipopolysaccharide biosynthesis
Lipopolysaccharide biosynthesis proteins	41416	65691	Metabolism; Glycan Biosynthesis and Metabolism; Lipopolysaccharide biosynthesis proteins
Lysine biosynthesis	74381	124610	Metabolism; Amino Acid Metabolism; Lysine biosynthesis
Lysine degradation	34565	44700	Metabolism; Amino Acid Metabolism; Lysine degradation
Lysosome	1920	1288	Cellular Processes; Transport and Catabolism; Lysosome
MAPK signaling pathway - yeast	5041	8192	Environmental Information Processing; Signal Transduction; MAPK signaling pathway - yeast
Meiosis - yeast	14435	28764	Cellular Processes; Cell Growth and Death; Meiosis - yeast
Melanogenesis	6	4	Organismal Systems; Endocrine System; Melanogenesis
Membrane and intracellular structural molecules	82220	130344	Unclassified; Cellular Processes and Signaling; Membrane and intracellular structural molecules
Metabolism of cofactors and vitamins	24658	40463	Unclassified; Metabolism; Metabolism of cofactors and vitamins
Metabolism of xenobiotics by cytochrome P450	37711	63773	Metabolism; Xenobiotics Biodegradation and Metabolism; Metabolism of xenobiotics by

			cytochrome P450
Methane metabolism	133705	233387	Metabolism; Energy Metabolism; Methane metabolism
Mineral absorption	10193	20938	Organismal Systems; Digestive System; Mineral absorption
Mismatch repair	89243	155641	Genetic Information Processing; Replication and Repair; Mismatch repair
N-Glycan biosynthesis	10937	21439	Metabolism; Glycan Biosynthesis and Metabolism; N-Glycan biosynthesis
NOD-like receptor signaling pathway	4187	7865	Organismal Systems; Immune System; NOD-like receptor signaling pathway
Naphthalene degradation	26464	42002	Metabolism; Xenobiotics Biodegradation and Metabolism; Naphthalene degradation
Neuroactive ligand-receptor interaction	0	1	Environmental Information Processing; Signaling Molecules and Interaction; Neuroactive ligand-receptor interaction
Nicotinate and nicotinamide metabolism	55299	94627	Metabolism; Metabolism of Cofactors and Vitamins; Nicotinate and nicotinamide metabolism
Nitrogen metabolism	82916	137576	Metabolism; Energy Metabolism; Nitrogen metabolism
Nitrotoluene degradation	7098	9748	Metabolism; Xenobiotics Biodegradation and Metabolism; Nitrotoluene degradation
Non-homologous end-joining	2095	1907	Genetic Information Processing; Replication and Repair; Non-homologous end-joining
Novobiocin biosynthesis	14714	24512	Metabolism; Biosynthesis of Other Secondary Metabolites; Novobiocin biosynthesis
Nucleotide excision repair	38855	66333	Genetic Information Processing; Replication and Repair; Nucleotide excision repair
Nucleotide metabolism	2204	1725	Unclassified; Metabolism; Nucleotide metabolism
One carbon pool by folate	62113	105729	Metabolism; Metabolism of Cofactors and Vitamins; One carbon pool by folate
Other glycan degradation	7500	9678	Metabolism; Glycan Biosynthesis and Metabolism; Other glycan degradation
Other ion-coupled transporters	130097	204989	Unclassified; Cellular Processes and Signaling; Other ion-coupled transporters
Other transporters	37610	63436	Unclassified; Cellular Processes and Signaling; Other transporters
Others	170641	305270	Unclassified; Metabolism; Others
Oxidative phosphorylation	201736	353921	Metabolism; Energy Metabolism; Oxidative phosphorylation
PPAR signaling pathway	23503	39740	Organismal Systems; Endocrine System; PPAR signaling pathway
Pancreatic secretion	0	1	Organismal Systems; Digestive System; Pancreatic secretion
Pantothenate and CoA biosynthesis	79612	139859	Metabolism; Metabolism of Cofactors and Vitamins; Pantothenate and CoA biosynthesis
Parkinson's disease	4880	4983	Human Diseases; Neurodegenerative Diseases; Parkinson's disease
Pathogenic Escherichia coli infection	160	0	Human Diseases; Infectious Diseases; Pathogenic Escherichia coli infection
Pathways in cancer	10713	17835	Human Diseases; Cancers; Pathways in cancer
Penicillin and cephalosporin biosynthesis	5170	8220	Metabolism; Biosynthesis of Other Secondary Metabolites; Penicillin and cephalosporin biosynthesis
Pentose and glucuronate interconversions	40569	61842	Metabolism; Carbohydrate Metabolism; Pentose and glucuronate interconversions
Pentose phosphate pathway	104527	184723	Metabolism; Carbohydrate Metabolism; Pentose phosphate pathway
Peptidases	265375	480417	Metabolism; Enzyme Families; Peptidases
Peptidoglycan biosynthesis	88822	154227	Metabolism; Glycan Biosynthesis and Metabolism; Peptidoglycan biosynthesis
Peroxisome	31088	50191	Cellular Processes; Transport and Catabolism; Peroxisome
Pertussis	6876	9570	Human Diseases; Infectious Diseases; Pertussis
Phenylalanine metabolism	34661	53462	Metabolism; Amino Acid Metabolism; Phenylalanine metabolism
Phenylalanine, tyrosine and tryptophan biosynthesis	92424	161433	Metabolism; Amino Acid Metabolism; Phenylalanine, tyrosine and tryptophan biosynthesis
Phenylpropanoid biosynthesis	17314	30723	Metabolism; Biosynthesis of Other Secondary Metabolites; Phenylpropanoid biosynthesis
Phosphatidylinositol signaling system	17335	31423	Environmental Information Processing; Signal Transduction; Phosphatidylinositol signaling system

Phosphonate and phosphinate metabolism	2757	1976	Metabolism; Metabolism of Other Amino Acids; Phosphonate and phosphinate metabolism
Phosphotransferase system (PTS)	16107	11879	Environmental Information Processing; Membrane Transport; Phosphotransferase system (PTS)
Photosynthesis	228365	470717	Metabolism; Energy Metabolism; Photosynthesis
Photosynthesis - antenna proteins	54655	116450	Metabolism; Energy Metabolism; Photosynthesis - antenna proteins
Photosynthesis proteins	287370	594908	Metabolism; Energy Metabolism; Photosynthesis proteins
Plant-pathogen interaction	11568	18216	Organismal Systems; Environmental Adaptation; Plant-pathogen interaction
Polycyclic aromatic hydrocarbon degradation	17252	31027	Metabolism; Xenobiotics Biodegradation and Metabolism; Polycyclic aromatic hydrocarbon degradation
Polyketide sugar unit biosynthesis	27478	51420	Metabolism; Metabolism of Terpenoids and Polyketides; Polyketide sugar unit biosynthesis
Pores ion channels	87876	149032	Unclassified; Cellular Processes and Signaling; Pores ion channels
Porphyrin and chlorophyll metabolism	230681	442540	Metabolism; Metabolism of Cofactors and Vitamins; Porphyrin and chlorophyll metabolism
Prenyltransferases	64096	120581	Metabolism; Metabolism of Terpenoids and Polyketides; Prenyltransferases
Primary bile acid biosynthesis	502	406	Metabolism; Lipid Metabolism; Primary bile acid biosynthesis
Primary immunodeficiency	4757	8017	Human Diseases; Immune System Diseases; Primary immunodeficiency
Prion diseases	651	446	Human Diseases; Neurodegenerative Diseases; Prion diseases
Progesterone-mediated oocyte maturation	4179	7862	Organismal Systems; Endocrine System; Progesterone-mediated oocyte maturation
Propanoate metabolism	76329	106662	Metabolism; Carbohydrate Metabolism; Propanoate metabolism
Prostate cancer	4180	7863	Human Diseases; Cancers; Prostate cancer
Proteasome	4398	8092	Genetic Information Processing; Folding, Sorting and Degradation; Proteasome
Protein digestion and absorption	393	243	Organismal Systems; Digestive System; Protein digestion and absorption
Protein export	72680	125143	Genetic Information Processing; Folding, Sorting and Degradation; Protein export
Protein folding and associated processing	128299	230540	Unclassified; Genetic Information Processing; Protein folding and associated processing
Protein kinases	83268	156300	Metabolism; Enzyme Families; Protein kinases
Protein processing in endoplasmic reticulum	14788	29109	Genetic Information Processing; Folding, Sorting and Degradation; Protein processing in endoplasmic reticulum
Proximal tubule bicarbonate reclamation	4375	7662	Organismal Systems; Excretory System; Proximal tubule bicarbonate reclamation
Purine metabolism	270150	464741	Metabolism; Nucleotide Metabolism; Purine metabolism
Pyrimidine metabolism	177101	302688	Metabolism; Nucleotide Metabolism; Pyrimidine metabolism
Pyruvate metabolism	145775	248207	Metabolism; Carbohydrate Metabolism; Pyruvate metabolism
RIG-I-like receptor signaling pathway	251	298	Organismal Systems; Immune System; RIG-I-like receptor signaling pathway
RNA degradation	75528	138221	Genetic Information Processing; Folding, Sorting and Degradation; RNA degradation
RNA polymerase	22100	39868	Genetic Information Processing; Transcription; RNA polymerase
RNA transport	11843	21857	Genetic Information Processing; Translation; RNA transport
Renal cell carcinoma	4668	8237	Human Diseases; Cancers; Renal cell carcinoma
Renin-angiotensin system	140	122	Organismal Systems; Endocrine System; Renin-angiotensin system
Replication, recombination and repair proteins	129603	234334	Unclassified; Genetic Information Processing; Replication, recombination and repair proteins
Restriction enzyme	15217	28935	Unclassified; Genetic Information Processing; Restriction enzyme
Retinol metabolism	16979	30404	Metabolism; Metabolism of Cofactors and Vitamins; Retinol metabolism
Riboflavin metabolism	43445	77306	Metabolism; Metabolism of Cofactors and Vitamins; Riboflavin metabolism
Ribosome	258013	451741	Genetic Information Processing; Translation;

			Ribosome
Ribosome Biogenesis	145636	247248	Genetic Information Processing; Translation; Ribosome Biogenesis
Ribosome biogenesis in eukaryotes	5256	8638	Genetic Information Processing; Translation; Ribosome biogenesis in eukaryotes
Secondary bile acid biosynthesis	135	122	Metabolism; Lipid Metabolism; Secondary bile acid biosynthesis
Secretion system	221103	359887	Environmental Information Processing; Membrane Transport; Secretion system
Selenocompound metabolism	44420	73573	Metabolism; Metabolism of Other Amino Acids; Selenocompound metabolism
Sesquiterpenoid biosynthesis	1	4	Metabolism; Metabolism of Terpenoids and Polyketides; Sesquiterpenoid biosynthesis
Shigellosis	92	16	Human Diseases; Infectious Diseases; Shigellosis
Signal transduction mechanisms	79152	143503	Unclassified; Cellular Processes and Signaling; Signal transduction mechanisms
Small cell lung cancer	1866	1736	Human Diseases; Cancers; Small cell lung cancer
Sphingolipid metabolism	6060	8815	Metabolism; Lipid Metabolism; Sphingolipid metabolism
Sporulation	9485	14675	Unclassified; Cellular Processes and Signaling; Sporulation
Staphylococcus aureus infection	643	366	Human Diseases; Infectious Diseases; Staphylococcus aureus infection
Starch and sucrose metabolism	108082	196388	Metabolism; Carbohydrate Metabolism; Starch and sucrose metabolism
Steroid biosynthesis	6814	13908	Metabolism; Lipid Metabolism; Steroid biosynthesis
Steroid hormone biosynthesis	4213	7583	Metabolism; Lipid Metabolism; Steroid hormone biosynthesis
Stilbenoid, diarylheptanoid and gingerol biosynthesis	7296	14404	Metabolism; Biosynthesis of Other Secondary Metabolites; Stilbenoid, diarylheptanoid and gingerol biosynthesis
Streptomycin biosynthesis	48716	89980	Metabolism; Biosynthesis of Other Secondary Metabolites; Streptomycin biosynthesis
Styrene degradation	8029	10382	Metabolism; Xenobiotics Biodegradation and Metabolism; Styrene degradation
Sulfur metabolism	45658	78121	Metabolism; Energy Metabolism; Sulfur metabolism
Sulfur relay system	55338	99489	Genetic Information Processing; Folding, Sorting and Degradation; Sulfur relay system
Synthesis and degradation of ketone bodies	6899	5138	Metabolism; Lipid Metabolism; Synthesis and degradation of ketone bodies
Systemic lupus erythematosus	12	11	Human Diseases; Immune System Diseases; Systemic lupus erythematosus
Taurine and hypotaurine metabolism	14814	24170	Metabolism; Metabolism of Other Amino Acids; Taurine and hypotaurine metabolism
Terpenoid backbone biosynthesis	70590	123887	Metabolism; Metabolism of Terpenoids and Polyketides; Terpenoid backbone biosynthesis
Tetracycline biosynthesis	21240	37732	Metabolism; Metabolism of Terpenoids and Polyketides; Tetracycline biosynthesis
Thiamine metabolism	52865	92406	Metabolism; Metabolism of Cofactors and Vitamins; Thiamine metabolism
Toluene degradation	22548	36300	Metabolism; Xenobiotics Biodegradation and Metabolism; Toluene degradation
Toxoplasmosis	1865	1736	Human Diseases; Infectious Diseases; Toxoplasmosis
Transcription factors	145561	221822	Genetic Information Processing; Transcription; Transcription factors
Transcription machinery	94171	162229	Genetic Information Processing; Transcription; Transcription machinery
Transcription related proteins	532	426	Unclassified; Genetic Information Processing; Transcription related proteins
Translation factors	59437	104928	Genetic Information Processing; Translation; Translation factors
Translation proteins	112230	197169	Unclassified; Genetic Information Processing; Translation proteins
Transporters	760110	1321677	Environmental Information Processing; Membrane Transport; Transporters
Tropane, piperidine and pyridine alkaloid biosynthesis	14836	24757	Metabolism; Biosynthesis of Other Secondary Metabolites; Tropane, piperidine and pyridine alkaloid biosynthesis
Tryptophan metabolism	46269	62257	Metabolism; Amino Acid Metabolism; Tryptophan metabolism
Tuberculosis	41942	81064	Human Diseases; Infectious Diseases; Tuberculosis

Two-component system	286219	481741	Environmental Information Processing; Signal Transduction; Two-component system
Type I diabetes mellitus	8354	15407	Human Diseases; Metabolic Diseases; Type I diabetes mellitus
Type II diabetes mellitus	11199	21584	Human Diseases; Metabolic Diseases; Type II diabetes mellitus
Tyrosine metabolism	57894	96879	Metabolism; Amino Acid Metabolism; Tyrosine metabolism
Ubiquinone and other terpenoid-quinone biosynthesis	80224	151385	Metabolism; Metabolism of Cofactors and Vitamins; Ubiquinone and other terpenoid-quinone biosynthesis
Ubiquitin system	3646	7204	Genetic Information Processing; Folding, Sorting and Degradation; Ubiquitin system
VEGF signaling pathway	1	0	Environmental Information Processing; Signal Transduction; VEGF signaling pathway
Valine, leucine and isoleucine biosynthesis	95958	166253	Metabolism; Amino Acid Metabolism; Valine, leucine and isoleucine biosynthesis
Valine, leucine and isoleucine degradation	65052	86526	Metabolism; Amino Acid Metabolism; Valine, leucine and isoleucine degradation
Various types of N-glycan biosynthesis	4	1	Metabolism; Glycan Biosynthesis and Metabolism; Various types of N-glycan biosynthesis
Vasopressin-regulated water reabsorption	3	0	Organismal Systems; Excretory System; Vasopressin-regulated water reabsorption
Vibrio cholerae infection	38	27	Human Diseases; Infectious Diseases; Vibrio cholerae infection
Vibrio cholerae pathogenic cycle	19574	36650	Human Diseases; Infectious Diseases; Vibrio cholerae pathogenic cycle
Viral myocarditis	1865	1736	Human Diseases; Cardiovascular Diseases; Viral myocarditis
Vitamin B6 metabolism	23098	39267	Metabolism; Metabolism of Cofactors and Vitamins; Vitamin B6 metabolism
Xylene degradation	8684	15342	Metabolism; Xenobiotics Biodegradation and Metabolism; Xylene degradation
Zeatin biosynthesis	4749	8264	Metabolism; Metabolism of Terpenoids and Polyketides; Zeatin biosynthesis
alpha-Linolenic acid metabolism	4784	8076	Metabolism; Lipid Metabolism; alpha-Linolenic acid metabolism
beta-Alanine metabolism	42235	63073	Metabolism; Metabolism of Other Amino Acids; beta-Alanine metabolism
beta-Lactam resistance	4796	7900	Metabolism; Biosynthesis of Other Secondary Metabolites; beta-Lactam resistance
mRNA surveillance pathway	5	0	Genetic Information Processing; Translation; mRNA surveillance pathway
p53 signaling pathway	1871	1741	Cellular Processes; Cell Growth and Death; p53 signaling pathway

Table S2 Results of PICRUSt at level 3 KEGG Orthology using the V3-V4 amplification regions.

OTU ID	B1(V3V4)	A1(V3V4)	KEGG Pathways
1,1,1-Trichloro-2,2-bis(4-chlorophenyl)ethane (DDT) degradation	70	47	Metabolism; Xenobiotics Biodegradation and Metabolism;1,1,1-Trichloro-2,2-bis(4-chlorophenyl) ethane (DDT) degradation
ABC transporters	838889	879449	Environmental Information Processing; Membrane Transport; ABC transporters
Adipocytokine signaling pathway	17270	16649	Organismal Systems; Endocrine System; Adipocytokine signaling pathway
African trypanosomiasis	5354	2931	Human Diseases; Infectious Diseases; African trypanosomiasis
Alanine, aspartate and glutamate metabolism	219137	212031	Metabolism; Amino Acid Metabolism; Alanine, aspartate and glutamate metabolism
Aldosterone-regulated sodium reabsorption	4	4	Organismal Systems; Excretory System; Aldosterone-regulated sodium reabsorption
Alzheimer's disease	32132	18582	Human Diseases; Neurodegenerative Diseases; Alzheimer's disease
Amino acid metabolism	64578	68137	Unclassified; Metabolism; Amino acid metabolism
Amino acid related enzymes	340779	299913	Metabolism; Amino Acid Metabolism; Amino acid related enzymes
Amino sugar and nucleotide sugar metabolism	272091	275350	Metabolism; Carbohydrate Metabolism; Amino sugar and nucleotide sugar metabolism
Aminoacyl-tRNA biosynthesis	292026	250991	Genetic Information Processing; Translation; Aminoacyl-tRNA biosynthesis
Aminobenzoate degradation	84289	67494	Metabolism; Xenobiotics Biodegradation and Metabolism; Aminobenzoate degradation
Amoebiasis	5517	6479	Human Diseases; Infectious Diseases; Amoebiasis
Amyotrophic lateral sclerosis (ALS)	13843	6940	Human Diseases; Neurodegenerative Diseases; Amyotrophic lateral sclerosis (ALS)
Antigen processing and presentation	8263	7823	Organismal Systems; Immune System; Antigen processing and presentation
Apoptosis	7519	2955	Cellular Processes; Cell Growth and Death; Apoptosis
Arachidonic acid metabolism	19823	17862	Metabolism; Lipid Metabolism; Arachidonic acid metabolism
Arginine and proline metabolism	325255	316763	Metabolism; Amino Acid Metabolism; Arginine and proline metabolism
Ascorbate and aldarate metabolism	38742	37090	Metabolism; Carbohydrate Metabolism; Ascorbate and aldarate metabolism
Atrazine degradation	20412	21850	Metabolism; Xenobiotics Biodegradation and Metabolism; Atrazine degradation
Bacterial chemotaxis	103528	71535	Cellular Processes; Cell Motility; Bacterial chemotaxis
Bacterial invasion of epithelial cells	4586	5628	Human Diseases; Infectious Diseases; Bacterial invasion of epithelial cells
Bacterial motility proteins	363332	327790	Cellular Processes; Cell Motility; Bacterial motility proteins
Bacterial secretion system	215325	164030	Environmental Information Processing; Membrane Transport; Bacterial secretion system
Bacterial toxins	17418	16794	Environmental Information Processing; Signaling Molecules and Interaction; Bacterial toxins
Basal transcription factors	471	376	Genetic Information Processing; Transcription; Basal transcription factors
Base excision repair	108354	97076	Genetic Information Processing; Replication and Repair; Base excision repair
Benzoate degradation	95262	69926	Metabolism; Xenobiotics Biodegradation and Metabolism; Benzoate degradation
Betalain biosynthesis	96	73	Metabolism; Biosynthesis of Other Secondary Metabolites; Betalain biosynthesis
Bile secretion	505	264	Organismal Systems; Digestive System; Bile secretion
Biosynthesis and biodegradation of secondary metabolites	20858	21725	Unclassified; Metabolism; Biosynthesis and biodegradation of secondary metabolites
Biosynthesis of 12-, 14- and 16-membered macrolides	24	42	Metabolism; Metabolism of Terpenoids and Polyketides; Biosynthesis of 12-, 14- and 16-membered macrolides
Biosynthesis of ansamycins	11427	11245	Metabolism; Metabolism of Terpenoids and Polyketides; Biosynthesis of ansamycins
Biosynthesis of siderophore group	11936	13077	Metabolism; Metabolism of Terpenoids and

nonribosomal peptides			Polyketides; Biosynthesis of siderophore group nonribosomal peptides
Biosynthesis of type II polyketide backbone	27	52	Metabolism; Metabolism of Terpenoids and Polyketides; Biosynthesis of type II polyketide backbone
Biosynthesis of type II polyketide products	43	76	Metabolism; Metabolism of Terpenoids and Polyketides; Biosynthesis of type II polyketide products
Biosynthesis of unsaturated fatty acids	60392	58599	Metabolism; Lipid Metabolism; Biosynthesis of unsaturated fatty acids
Biosynthesis of vancomycin group antibiotics	13808	14385	Metabolism; Metabolism of Terpenoids and Polyketides; Biosynthesis of vancomycin group antibiotics
Biotin metabolism	40972	39829	Metabolism; Metabolism of Cofactors and Vitamins; Biotin metabolism
Bisphenol degradation	17194	14009	Metabolism; Xenobiotics Biodegradation and Metabolism; Bisphenol degradation
Bladder cancer	1709	1305	Human Diseases; Cancers; Bladder cancer
Butanoate metabolism	203754	163909	Metabolism; Carbohydrate Metabolism; Butanoate metabolism
Butirosin and neomycin biosynthesis	13108	14191	Metabolism; Biosynthesis of Other Secondary Metabolites; Butirosin and neomycin biosynthesis
C5-Branched dibasic acid metabolism	72014	65403	Metabolism; Carbohydrate Metabolism; C5-Branched dibasic acid metabolism
CAM ligands	1	0	Environmental Information Processing; Signaling Molecules and Interaction; CAM ligands
Caffeine metabolism	454	388	Metabolism; Biosynthesis of Other Secondary Metabolites; Caffeine metabolism
Calcium signaling pathway	4439	5578	Environmental Information Processing; Signal Transduction; Calcium signaling pathway
Caprolactam degradation	40077	28226	Metabolism; Xenobiotics Biodegradation and Metabolism; Caprolactam degradation
Carbohydrate digestion and absorption	5547	6819	Organismal Systems; Digestive System; Carbohydrate digestion and absorption
Carbohydrate metabolism	54434	58884	Unclassified; Metabolism; Carbohydrate metabolism
Carbon fixation in photosynthetic organisms	169724	167170	Metabolism; Energy Metabolism; Carbon fixation in photosynthetic organisms
Carbon fixation pathways in prokaryotes	240234	204720	Metabolism; Energy Metabolism; Carbon fixation pathways in prokaryotes
Cardiac muscle contraction	13187	5807	Organismal Systems; Circulatory System; Cardiac muscle contraction
Carotenoid biosynthesis	43855	47810	Metabolism; Metabolism of Terpenoids and Polyketides; Carotenoid biosynthesis
Cell cycle - Caulobacter	162180	132310	Cellular Processes; Cell Growth and Death; Cell cycle - Caulobacter
Cell division	20708	18449	Unclassified; Cellular Processes and Signaling; Cell division
Cell motility and secretion	73507	68411	Unclassified; Cellular Processes and Signaling; Cell motility and secretion
Cellular antigens	19049	17323	Environmental Information Processing; Signaling Molecules and Interaction; Cellular antigens
Chagas disease (American trypanosomiasis)	5472	2641	Human Diseases; Infectious Diseases; Chagas disease (American trypanosomiasis)
Chaperones and folding catalysts	290948	275682	Genetic Information Processing; Folding, Sorting and Degradation; Chaperones and folding catalysts
Chloroalkane and chloroalkene degradation	66939	61879	Metabolism; Xenobiotics Biodegradation and Metabolism; Chloroalkane and chloroalkene degradation
Chlorocyclohexane and chlorobenzene degradation	28978	25240	Metabolism; Xenobiotics Biodegradation and Metabolism; Chlorocyclohexane and chlorobenzene degradation
Chromosome	396231	371779	Genetic Information Processing; Replication and Repair; Chromosome
Chronic myeloid leukemia	2	1	Human Diseases; Cancers; Chronic myeloid leukemia
Circadian rhythm - plant	1841	887	Organismal Systems; Environmental Adaptation; Circadian rhythm - plant
Citrate cycle (TCA cycle)	183081	140328	Metabolism; Carbohydrate Metabolism; Citrate cycle (TCA cycle)

Clavulanic acid biosynthesis	23	48	Metabolism; Biosynthesis of Other Secondary Metabolites; Clavulanic acid biosynthesis
Colorectal cancer	7322	2782	Human Diseases; Cancers; Colorectal cancer
Cyanoamino acid metabolism	63074	59639	Metabolism; Metabolism of Other Amino Acids; Cyanoamino acid metabolism
Cysteine and methionine metabolism	195509	190500	Metabolism; Amino Acid Metabolism; Cysteine and methionine metabolism
Cytochrome P450	400	64	Metabolism; Enzyme Families; Cytochrome P450
Cytokine receptors	0	1	Environmental Information Processing; Signaling Molecules and Interaction; Cytokine receptors
Cytokine-cytokine receptor interaction	0	1	Environmental Information Processing; Signaling Molecules and Interaction; Cytokine-cytokine receptor interaction
Cytoskeleton proteins	87410	80737	Cellular Processes; Cell Motility; Cytoskeleton proteins
D-Alanine metabolism	18794	18316	Metabolism; Metabolism of Other Amino Acids; D-Alanine metabolism
D-Arginine and D-ornithine metabolism	617	389	Metabolism; Metabolism of Other Amino Acids; D-Arginine and D-ornithine metabolism
D-Glutamine and D-glutamate metabolism	35762	33043	Metabolism; Metabolism of Other Amino Acids; D-Glutamine and D-glutamate metabolism
DNA repair and recombination proteins	692121	629127	Genetic Information Processing; Replication and Repair; DNA repair and recombination proteins
DNA replication	145530	122119	Genetic Information Processing; Replication and Repair; DNA replication
DNA replication proteins	262774	226574	Genetic Information Processing; Replication and Repair; DNA replication proteins
Dioxin degradation	22419	18175	Metabolism; Xenobiotics Biodegradation and Metabolism; Dioxin degradation
Drug metabolism - cytochrome P450	71891	65630	Metabolism; Xenobiotics Biodegradation and Metabolism; Drug metabolism - cytochrome P450
Drug metabolism - other enzymes	39881	36227	Metabolism; Xenobiotics Biodegradation and Metabolism; Drug metabolism - other enzymes
ECM-receptor interaction	1	0	Environmental Information Processing; Signaling Molecules and Interaction; ECM-receptor interaction
Electron transfer carriers	8575	10259	Unclassified; Cellular Processes and Signaling; Electron transfer carriers
Endocytosis	43	30	Cellular Processes; Transport and Catabolism; Endocytosis
Energy metabolism	172951	156130	Unclassified; Metabolism; Energy metabolism
Epithelial cell signaling in Helicobacter pylori infection	13213	14744	Human Diseases; Infectious Diseases; Epithelial cell signaling in Helicobacter pylori infection
Ether lipid metabolism	793	943	Metabolism; Lipid Metabolism; Ether lipid metabolism
Ethylbenzene degradation	15100	12370	Metabolism; Xenobiotics Biodegradation and Metabolism; Ethylbenzene degradation
Fatty acid biosynthesis	129976	117563	Metabolism; Lipid Metabolism; Fatty acid biosynthesis
Fatty acid elongation in mitochondria	3	1	Metabolism; Lipid Metabolism; Fatty acid elongation in mitochondria
Fatty acid metabolism	152533	131077	Metabolism; Lipid Metabolism; Fatty acid metabolism
Fc gamma R-mediated phagocytosis	43	30	Organismal Systems; Immune System; Fc gamma R-mediated phagocytosis
Flagellar assembly	93348	59621	Cellular Processes; Cell Motility; Flagellar assembly
Flavone and flavonol biosynthesis	63	48	Metabolism; Biosynthesis of Other Secondary Metabolites; Flavone and flavonol biosynthesis
Flavonoid biosynthesis	16066	18074	Metabolism; Biosynthesis of Other Secondary Metabolites; Flavonoid biosynthesis
Fluorobenzoate degradation	20873	17119	Metabolism; Xenobiotics Biodegradation and Metabolism; Fluorobenzoate degradation
Focal adhesion	1	0	Cellular Processes; Cell Communication; Focal adhesion
Folate biosynthesis	117372	114805	Metabolism; Metabolism of Cofactors and Vitamins; Folate biosynthesis
Fructose and mannose metabolism	164812	171876	Metabolism; Carbohydrate Metabolism; Fructose and mannose metabolism
Function unknown	402812	383841	Unclassified; Poorly Characterized; Function unknown

G protein-coupled receptors	15	12	Environmental Information Processing; Signaling Molecules and Interaction; G protein-coupled receptors
Galactose metabolism	87133	84438	Metabolism; Carbohydrate Metabolism; Galactose metabolism
General function prediction only	951042	935793	Unclassified; Poorly Characterized; General function prediction only
Geraniol degradation	52264	42014	Metabolism; Metabolism of Terpenoids and Polyketides; Geraniol degradation
Germination	1269	977	Unclassified; Cellular Processes and Signaling; Germination
Glutamatergic synapse	24372	23858	Organismal Systems; Nervous System; Glutamatergic synapse
Glutathione metabolism	144657	131061	Metabolism; Metabolism of Other Amino Acids; Glutathione metabolism
Glycan biosynthesis and metabolism	17335	18311	Unclassified; Metabolism; Glycan biosynthesis and metabolism
Glycerolipid metabolism	83021	83602	Metabolism; Lipid Metabolism; Glycerolipid metabolism
Glycerophospholipid metabolism	125471	110153	Metabolism; Lipid Metabolism; Glycerophospholipid metabolism
Glycine, serine and threonine metabolism	205820	182951	Metabolism; Amino Acid Metabolism; Glycine, serine and threonine metabolism
Glycolysis / Gluconeogenesis	286483	277910	Metabolism; Carbohydrate Metabolism; Glycolysis / Gluconeogenesis
Glycosaminoglycan degradation	4258	2557	Metabolism; Glycan Biosynthesis and Metabolism; Glycosaminoglycan degradation
Glycosphingolipid biosynthesis - ganglio series	2743	1820	Metabolism; Glycan Biosynthesis and Metabolism; Glycosphingolipid biosynthesis - ganglio series
Glycosphingolipid biosynthesis - globo series	4632	3320	Metabolism; Glycan Biosynthesis and Metabolism; Glycosphingolipid biosynthesis - globo series
Glycosphingolipid biosynthesis - lacto and neolacto series	69	24	Metabolism; Glycan Biosynthesis and Metabolism; Glycosphingolipid biosynthesis - lacto and neolacto series
Glycosyltransferases	126282	136960	Metabolism; Glycan Biosynthesis and Metabolism; Glycosyltransferases
Glyoxylate and dicarboxylate metabolism	172170	146158	Metabolism; Carbohydrate Metabolism; Glyoxylate and dicarboxylate metabolism
GnRH signaling pathway	43	30	Organismal Systems; Endocrine System; GnRH signaling pathway
Hematopoietic cell lineage	18	5	Organismal Systems; Immune System; Hematopoietic cell lineage
Histidine metabolism	121264	120294	Metabolism; Amino Acid Metabolism; Histidine metabolism
Homologous recombination	209948	184868	Genetic Information Processing; Replication and Repair; Homologous recombination
Huntington's disease	38916	25207	Human Diseases; Neurodegenerative Diseases; Huntington's disease
Hypertrophic cardiomyopathy (HCM)	430	102	Human Diseases; Cardiovascular Diseases; Hypertrophic cardiomyopathy (HCM)
Indole alkaloid biosynthesis	89	49	Metabolism; Biosynthesis of Other Secondary Metabolites; Indole alkaloid biosynthesis
Influenza A	7323	2788	Human Diseases; Infectious Diseases; Influenza A
Inorganic ion transport and metabolism	65866	58334	Unclassified; Cellular Processes and Signaling; Inorganic ion transport and metabolism
Inositol phosphate metabolism	42404	34881	Metabolism; Carbohydrate Metabolism; Inositol phosphate metabolism
Insulin signaling pathway	32083	37884	Organismal Systems; Endocrine System; Insulin signaling pathway
Ion channels	7688	8267	Environmental Information Processing; Signaling Molecules and Interaction; Ion channels
Isoflavonoid biosynthesis	19	15	Metabolism; Biosynthesis of Other Secondary Metabolites; Isoflavonoid biosynthesis
Isoquinoline alkaloid biosynthesis	13536	11505	Metabolism; Biosynthesis of Other Secondary Metabolites; Isoquinoline alkaloid biosynthesis
Leishmaniasis	2	1	Human Diseases; Infectious Diseases; Leishmaniasis
Limonene and pinene degradation	59095	47210	Metabolism; Metabolism of Terpenoids and Polyketides; Limonene and pinene degradation
Linoleic acid metabolism	6771	4725	Metabolism; Lipid Metabolism; Linoleic acid metabolism

Lipid biosynthesis proteins	188136	171072	Metabolism; Lipid Metabolism; Lipid biosynthesis proteins
Lipid metabolism	41899	42339	Unclassified; Metabolism; Lipid metabolism
Lipoic acid metabolism	29811	29544	Metabolism; Metabolism of Cofactors and Vitamins; Lipoic acid metabolism
Lipopolysaccharide biosynthesis	54674	62348	Metabolism; Glycan Biosynthesis and Metabolism; Lipopolysaccharide biosynthesis
Lipopolysaccharide biosynthesis proteins	72591	80689	Metabolism; Glycan Biosynthesis and Metabolism; Lipopolysaccharide biosynthesis proteins
Lysine biosynthesis	158531	138441	Metabolism; Amino Acid Metabolism; Lysine biosynthesis
Lysine degradation	83427	66072	Metabolism; Amino Acid Metabolism; Lysine degradation
Lysosome	6053	3894	Cellular Processes; Transport and Catabolism; Lysosome
MAPK signaling pathway - yeast	10780	9357	Environmental Information Processing; Signal Transduction; MAPK signaling pathway - yeast
Meiosis - yeast	22232	24613	Cellular Processes; Cell Growth and Death; Meiosis - yeast
Melanogenesis	15	26	Organismal Systems; Endocrine System; Melanogenesis
Membrane and intracellular structural molecules	179219	158735	Unclassified; Cellular Processes and Signaling; Membrane and intracellular structural molecules
Metabolism of cofactors and vitamins	46511	45497	Unclassified; Metabolism; Metabolism of cofactors and vitamins
Metabolism of xenobiotics by cytochrome P450	71415	65135	Metabolism; Xenobiotics Biodegradation and Metabolism; Metabolism of xenobiotics by cytochrome P450
Methane metabolism	248182	244492	Metabolism; Energy Metabolism; Methane metabolism
Mineral absorption	14719	17808	Organismal Systems; Digestive System; Mineral absorption
Mismatch repair	188496	165419	Genetic Information Processing; Replication and Repair; Mismatch repair
N-Glycan biosynthesis	17134	19039	Metabolism; Glycan Biosynthesis and Metabolism; N-Glycan biosynthesis
NOD-like receptor signaling pathway	8270	7828	Organismal Systems; Immune System; NOD-like receptor signaling pathway
Naphthalene degradation	51776	47715	Metabolism; Xenobiotics Biodegradation and Metabolism; Naphthalene degradation
Neuroactive ligand-receptor interaction	1	6	Environmental Information Processing; Signaling Molecules and Interaction; Neuroactive ligand-receptor interaction
Nicotinate and nicotinamide metabolism	105143	103644	Metabolism; Metabolism of Cofactors and Vitamins; Nicotinate and nicotinamide metabolism
Nitrogen metabolism	164600	157962	Metabolism; Energy Metabolism; Nitrogen metabolism
Nitrotoluene degradation	13763	12170	Metabolism; Xenobiotics Biodegradation and Metabolism; Nitrotoluene degradation
Non-homologous end-joining	6539	4349	Genetic Information Processing; Replication and Repair; Non-homologous end-joining
Notch signaling pathway	2	1	Environmental Information Processing; Signal Transduction; Notch signaling pathway
Novobiocin biosynthesis	30423	27623	Metabolism; Biosynthesis of Other Secondary Metabolites; Novobiocin biosynthesis
Nucleotide excision repair	82276	72431	Genetic Information Processing; Replication and Repair; Nucleotide excision repair
Nucleotide metabolism	5659	6101	Unclassified; Metabolism; Nucleotide metabolism
One carbon pool by folate	129143	114822	Metabolism; Metabolism of Cofactors and Vitamins; One carbon pool by folate
Other glycan degradation	17795	13911	Metabolism; Glycan Biosynthesis and Metabolism; Other glycan degradation
Other ion-coupled transporters	272268	257850	Unclassified; Cellular Processes and Signaling; Other ion-coupled transporters
Other transporters	72195	68939	Unclassified; Cellular Processes and Signaling; Other transporters
Others	302436	310513	Unclassified; Metabolism; Others
Oxidative phosphorylation	434504	372767	Metabolism; Energy Metabolism; Oxidative phosphorylation
PPAR signaling pathway	44272	41981	Organismal Systems; Endocrine System; PPAR signaling pathway

Pancreatic secretion	1	6	Organismal Systems; Digestive System; Pancreatic secretion
Pantothenate and CoA biosynthesis	148700	145692	Metabolism; Metabolism of Cofactors and Vitamins; Pantothenate and CoA biosynthesis
Parkinson's disease	20509	8591	Human Diseases; Neurodegenerative Diseases; Parkinson's disease
Pathogenic Escherichia coli infection	110	40	Human Diseases; Infectious Diseases; Pathogenic Escherichia coli infection
Pathways in cancer	25620	19289	Human Diseases; Cancers; Pathways in cancer
Penicillin and cephalosporin biosynthesis	10397	9938	Metabolism; Biosynthesis of Other Secondary Metabolites; Penicillin and cephalosporin biosynthesis
Pentose and glucuronate interconversions	84788	80204	Metabolism; Carbohydrate Metabolism; Pentose and glucuronate interconversions
Pentose phosphate pathway	192684	194832	Metabolism; Carbohydrate Metabolism; Pentose phosphate pathway
Peptidases	481219	485869	Metabolism; Enzyme Families; Peptidases
Peptidoglycan biosynthesis	181227	164775	Metabolism; Glycan Biosynthesis and Metabolism; Peptidoglycan biosynthesis
Peroxisome	63703	56107	Cellular Processes; Transport and Catabolism; Peroxisome
Pertussis	14438	15908	Human Diseases; Infectious Diseases; Pertussis
Phenylalanine metabolism	82779	67016	Metabolism; Amino Acid Metabolism; Phenylalanine metabolism
Phenylalanine, tyrosine and tryptophan biosynthesis	172279	170403	Metabolism; Amino Acid Metabolism; Phenylalanine, tyrosine and tryptophan biosynthesis
Phenylpropanoid biosynthesis	32495	32179	Metabolism; Biosynthesis of Other Secondary Metabolites; Phenylpropanoid biosynthesis
Phosphatidylinositol signaling system	33016	31348	Environmental Information Processing; Signal Transduction; Phosphatidylinositol signaling system
Phosphonate and phosphinate metabolism	8463	5549	Metabolism; Metabolism of Other Amino Acids; Phosphonate and phosphinate metabolism
Phosphotransferase system (PTS)	40933	42030	Environmental Information Processing; Membrane Transport; Phosphotransferase system (PTS)
Photosynthesis	347467	399733	Metabolism; Energy Metabolism; Photosynthesis
Photosynthesis - antenna proteins	75448	94795	Metabolism; Energy Metabolism; Photosynthesis - antenna proteins
Photosynthesis proteins	431529	502186	Metabolism; Energy Metabolism; Photosynthesis proteins
Plant-pathogen interaction	27376	22728	Organismal Systems; Environmental Adaptation; Plant-pathogen interaction
Polycyclic aromatic hydrocarbon degradation	32623	31446	Metabolism; Xenobiotics Biodegradation and Metabolism; Polycyclic aromatic hydrocarbon degradation
Polyketide sugar unit biosynthesis	45370	48483	Metabolism; Metabolism of Terpenoids and Polyketides; Polyketide sugar unit biosynthesis
Pores ion channels	168202	163600	Unclassified; Cellular Processes and Signaling; Pores ion channels
Porphyrin and chlorophyll metabolism	401803	411543	Metabolism; Metabolism of Cofactors and Vitamins; Porphyrin and chlorophyll metabolism
Prenyltransferases	117025	116055	Metabolism; Metabolism of Terpenoids and Polyketides; Prenyltransferases
Primary bile acid biosynthesis	1752	1179	Metabolism; Lipid Metabolism; Primary bile acid biosynthesis
Primary immunodeficiency	9788	9609	Human Diseases; Immune System Diseases; Primary immunodeficiency
Prion diseases	2415	1392	Human Diseases; Neurodegenerative Diseases; Prion diseases
Progesterone-mediated oocyte maturation	8263	7823	Organismal Systems; Endocrine System; Progesterone-mediated oocyte maturation
Propanoate metabolism	174537	141554	Metabolism; Carbohydrate Metabolism; Propanoate metabolism
Prostate cancer	8265	7825	Human Diseases; Cancers; Prostate cancer
Proteasome	8964	8502	Genetic Information Processing; Folding, Sorting and Degradation; Proteasome
Protein digestion and absorption	1394	697	Organismal Systems; Digestive System; Protein digestion and absorption
Protein export	158319	135281	Genetic Information Processing; Folding, Sorting and Degradation; Protein export

Protein folding and associated processing	237174	236150	Unclassified; Genetic Information Processing; Protein folding and associated processing
Protein kinases	142290	155333	Metabolism; Enzyme Families; Protein kinases
Protein processing in endoplasmic reticulum	24794	26557	Genetic Information Processing; Folding, Sorting and Degradation; Protein processing in endoplasmic reticulum
Proximal tubule bicarbonate reclamation	7384	8118	Organismal Systems; Excretory System; Proximal tubule bicarbonate reclamation
Purine metabolism	536136	499884	Metabolism; Nucleotide Metabolism; Purine metabolism
Pyrimidine metabolism	367614	330371	Metabolism; Nucleotide Metabolism; Pyrimidine metabolism
Pyruvate metabolism	289433	270586	Metabolism; Carbohydrate Metabolism; Pyruvate metabolism
RIG-I-like receptor signaling pathway	670	795	Organismal Systems; Immune System; RIG-I-like receptor signaling pathway
RNA degradation	142790	138098	Genetic Information Processing; Folding, Sorting and Degradation; RNA degradation
RNA polymerase	45147	40516	Genetic Information Processing; Transcription; RNA polymerase
RNA transport	18147	21758	Genetic Information Processing; Translation; RNA transport
Renal cell carcinoma	10031	8682	Human Diseases; Cancers; Renal cell carcinoma
Renin-angiotensin system	662	359	Organismal Systems; Endocrine System; Renin-angiotensin system
Replication, recombination and repair proteins	228823	232657	Unclassified; Genetic Information Processing; Replication, recombination and repair proteins
Restriction enzyme	25303	29140	Unclassified; Genetic Information Processing; Restriction enzyme
Retinol metabolism	30092	30355	Metabolism; Metabolism of Cofactors and Vitamins; Retinol metabolism
Riboflavin metabolism	82678	80536	Metabolism; Metabolism of Cofactors and Vitamins; Riboflavin metabolism
Ribosome	551217	476326	Genetic Information Processing; Translation; Ribosome
Ribosome Biogenesis	303239	277490	Genetic Information Processing; Translation; Ribosome Biogenesis
Ribosome biogenesis in eukaryotes	11629	10377	Genetic Information Processing; Translation; Ribosome biogenesis in eukaryotes
Secondary bile acid biosynthesis	424	311	Metabolism; Lipid Metabolism; Secondary bile acid biosynthesis
Secretion system	492930	425147	Environmental Information Processing; Membrane Transport; Secretion system
Selenocompound metabolism	88917	82130	Metabolism; Metabolism of Other Amino Acids; Selenocompound metabolism
Sesquiterpenoid biosynthesis	5	15	Metabolism; Metabolism of Terpenoids and Polyketides; Sesquiterpenoid biosynthesis
Shigellosis	91	26	Human Diseases; Infectious Diseases; Shigellosis
Signal transduction mechanisms	142990	147751	Unclassified; Cellular Processes and Signaling; Signal transduction mechanisms
Small cell lung cancer	7324	2783	Human Diseases; Cancers; Small cell lung cancer
Sphingolipid metabolism	13952	11201	Metabolism; Lipid Metabolism; Sphingolipid metabolism
Sporulation	17895	16181	Unclassified; Cellular Processes and Signaling; Sporulation
Staphylococcus aureus infection	2168	999	Human Diseases; Infectious Diseases; Staphylococcus aureus infection
Starch and sucrose metabolism	193195	202649	Metabolism; Carbohydrate Metabolism; Starch and sucrose metabolism
Steroid biosynthesis	9918	11736	Metabolism; Lipid Metabolism; Steroid biosynthesis
Steroid hormone biosynthesis	8688	7652	Metabolism; Lipid Metabolism; Steroid hormone biosynthesis
Stilbenoid, diarylheptanoid and gingerol biosynthesis	12084	12857	Metabolism; Biosynthesis of Other Secondary Metabolites; Stilbenoid, diarylheptanoid and gingerol biosynthesis
Streptomycin biosynthesis	85392	87414	Metabolism; Biosynthesis of Other Secondary Metabolites; Streptomycin biosynthesis
Styrene degradation	22341	15352	Metabolism; Xenobiotics Biodegradation and Metabolism; Styrene degradation
Sulfur metabolism	85484	83627	Metabolism; Energy Metabolism; Sulfur metabolism

Sulfur relay system	99072	102952	Genetic Information Processing; Folding, Sorting and Degradation; Sulfur relay system
Synthesis and degradation of ketone bodies	21597	13290	Metabolism; Lipid Metabolism; Synthesis and degradation of ketone bodies
Systemic lupus erythematosus	38	53	Human Diseases; Immune System Diseases; Systemic lupus erythematosus
Taurine and hypotaurine metabolism	30265	27589	Metabolism; Metabolism of Other Amino Acids; Taurine and hypotaurine metabolism
Terpenoid backbone biosynthesis	131995	128387	Metabolism; Metabolism of Terpenoids and Polyketides; Terpenoid backbone biosynthesis
Tetracycline biosynthesis	37687	37838	Metabolism; Metabolism of Terpenoids and Polyketides; Tetracycline biosynthesis
Thiamine metabolism	97560	95908	Metabolism; Metabolism of Cofactors and Vitamins; Thiamine metabolism
Toluene degradation	54172	44615	Metabolism; Xenobiotics Biodegradation and Metabolism; Toluene degradation
Toxoplasmosis	7323	2782	Human Diseases; Infectious Diseases; Toxoplasmosis
Transcription factors	310199	293563	Genetic Information Processing; Transcription; Transcription factors
Transcription machinery	196916	175360	Genetic Information Processing; Transcription; Transcription machinery
Transcription related proteins	1512	1611	Unclassified; Genetic Information Processing; Transcription related proteins
Translation factors	122577	109956	Genetic Information Processing; Translation; Translation factors
Translation proteins	217651	207481	Unclassified; Genetic Information Processing; Translation proteins
Transporters	1394356	1453129	Environmental Information Processing; Membrane Transport; Transporters
Tropane, piperidine and pyridine alkaloid biosynthesis	30345	28282	Metabolism; Biosynthesis of Other Secondary Metabolites; Tropane, piperidine and pyridine alkaloid biosynthesis
Tryptophan metabolism	110001	88400	Metabolism; Amino Acid Metabolism; Tryptophan metabolism
Tuberculosis	72339	73795	Human Diseases; Infectious Diseases; Tuberculosis
Two-component system	558228	538181	Environmental Information Processing; Signal Transduction; Two-component system
Type I diabetes mellitus	15408	14945	Human Diseases; Metabolic Diseases; Type I diabetes mellitus
Type II diabetes mellitus	17429	19735	Human Diseases; Metabolic Diseases; Type II diabetes mellitus
Tyrosine metabolism	112956	108059	Metabolism; Amino Acid Metabolism; Tyrosine metabolism
Ubiquinone and other terpenoid-quinone biosynthesis	147041	148142	Metabolism; Metabolism of Cofactors and Vitamins; Ubiquinone and other terpenoid-quinone biosynthesis
Ubiquitin system	5690	6969	Genetic Information Processing; Folding, Sorting and Degradation; Ubiquitin system
VEGF signaling pathway	2	1	Environmental Information Processing; Signal Transduction; VEGF signaling pathway
Valine, leucine and isoleucine biosynthesis	190332	178026	Metabolism; Amino Acid Metabolism; Valine, leucine and isoleucine biosynthesis
Valine, leucine and isoleucine degradation	164851	122760	Metabolism; Amino Acid Metabolism; Valine, leucine and isoleucine degradation
Various types of N-glycan biosynthesis	2	15	Metabolism; Glycan Biosynthesis and Metabolism; Various types of N-glycan biosynthesis
Vasopressin-regulated water reabsorption	1	3	Organismal Systems; Excretory System; Vasopressin-regulated water reabsorption
Vibrio cholerae infection	100	184	Human Diseases; Infectious Diseases; Vibrio cholerae infection
Vibrio cholerae pathogenic cycle	33025	36449	Human Diseases; Infectious Diseases; Vibrio cholerae pathogenic cycle
Viral myocarditis	7322	2782	Human Diseases; Cardiovascular Diseases; Viral myocarditis
Vitamin B6 metabolism	43697	43380	Metabolism; Metabolism of Cofactors and Vitamins; Vitamin B6 metabolism
Wnt signaling pathway	2	1	Environmental Information Processing; Signal Transduction; Wnt signaling pathway
Xylene degradation	19580	16081	Metabolism; Xenobiotics Biodegradation and Metabolism; Xylene degradation
Zeatin biosynthesis	10226	8755	Metabolism; Metabolism of Terpenoids and Polyketides; Zeatin biosynthesis

alpha-Linolenic acid metabolism	9586	9834	Metabolism; Lipid Metabolism; alpha-Linolenic acid metabolism
beta-Alanine metabolism	88952	79073	Metabolism; Metabolism of Other Amino Acids; beta-Alanine metabolism
beta-Lactam resistance	9277	8786	Metabolism; Biosynthesis of Other Secondary Metabolites; beta-Lactam resistance
mRNA surveillance pathway	14	3	Genetic Information Processing; Translation; mRNA surveillance pathway
p53 signaling pathway	7358	2808	Cellular Processes; Cell Growth and Death; p53 signaling pathway

Table S3 Results of PICRUSt at level 3 KEGG Orthology using the V4-V5 amplification regions

OTU ID	A1(V4V5)	B1(V4V5)	KEGG Pathways
1,1,1-Trichloro-2,2-bis(4-chlorophenyl)ethane (DDT) degradation	99	90	Metabolism; Xenobiotics Biodegradation and Metabolism; 1,1,1-Trichloro-2,2-bis(4-chlorophenyl)ethane (DDT) degradation
ABC transporters	1532954	1614032	Environmental Information Processing; Membrane Transport; ABC transporters
Adherens junction	1	0	Cellular Processes; Cell Communication; Adherens junction
Adipocytokine signaling pathway	30954	32807	Organismal Systems; Endocrine System; Adipocytokine signaling pathway
African trypanosomiasis	4760	6159	Human Diseases; Infectious Diseases; African trypanosomiasis
Alanine, aspartate and glutamate metabolism	382785	408174	Metabolism; Amino Acid Metabolism; Alanine, aspartate and glutamate metabolism
Aldosterone-regulated sodium reabsorption	7	3	Organismal Systems; Excretory System; Aldosterone-regulated sodium reabsorption
Alzheimer's disease	35441	41569	Human Diseases; Neurodegenerative Diseases; Alzheimer's disease
Amino acid metabolism	128081	129484	Unclassified; Metabolism; Amino acid metabolism
Amino acid related enzymes	560405	597026	Metabolism; Amino Acid Metabolism; Amino acid related enzymes
Amino sugar and nucleotide sugar metabolism	499939	521219	Metabolism; Carbohydrate Metabolism; Amino sugar and nucleotide sugar metabolism
Aminoacyl-tRNA biosynthesis	493082	512337	Genetic Information Processing; Translation; Aminoacyl-tRNA biosynthesis
Aminobenzoate degradation	111788	132106	Metabolism; Xenobiotics Biodegradation and Metabolism; Aminobenzoate degradation
Amoebiasis	13069	12649	Human Diseases; Infectious Diseases; Amoebiasis
Amyotrophic lateral sclerosis (ALS)	9667	15022	Human Diseases; Neurodegenerative Diseases; Amyotrophic lateral sclerosis (ALS)
Antigen processing and presentation	15963	16020	Organismal Systems; Immune System; Antigen processing and presentation
Apoptosis	5684	7710	Cellular Processes; Cell Growth and Death; Apoptosis
Arachidonic acid metabolism	31512	34818	Metabolism; Lipid Metabolism; Arachidonic acid metabolism
Arginine and proline metabolism	573772	605515	Metabolism; Amino Acid Metabolism; Arginine and proline metabolism
Arrhythmogenic right ventricular cardiomyopathy (ARVC)	1	0	Human Diseases; Cardiovascular Diseases; Arrhythmogenic right ventricular cardiomyopathy (ARVC)
Ascorbate and aldarate metabolism	53591	63226	Metabolism; Carbohydrate Metabolism; Ascorbate and aldarate metabolism
Atrazine degradation	41229	40986	Metabolism; Xenobiotics Biodegradation and Metabolism; Atrazine degradation
Bacterial chemotaxis	96359	142687	Cellular Processes; Cell Motility; Bacterial chemotaxis
Bacterial invasion of epithelial cells	12316	11754	Human Diseases; Infectious Diseases; Bacterial invasion of epithelial cells
Bacterial motility proteins	542976	635444	Cellular Processes; Cell Motility; Bacterial motility proteins
Bacterial secretion system	285842	329025	Environmental Information Processing; Membrane Transport; Bacterial secretion system
Bacterial toxins	30796	33408	Environmental Information Processing; Signaling Molecules and Interaction; Bacterial toxins
Basal transcription factors	427	672	Genetic Information Processing; Transcription; Basal transcription factors
Base excision repair	173734	188027	Genetic Information Processing; Replication and Repair; Base excision repair
Benzoate degradation	101475	131952	Metabolism; Xenobiotics Biodegradation and Metabolism; Benzoate degradation
Betalain biosynthesis	94	297	Metabolism; Biosynthesis of Other Secondary Metabolites; Betalain biosynthesis
Bile secretion	236	441	Organismal Systems; Digestive System; Bile secretion
Biosynthesis and biodegradation of secondary metabolites	32143	36392	Unclassified; Metabolism; Biosynthesis and biodegradation of secondary metabolites
Biosynthesis of 12-, 14- and 16-membered macrolides	15	21	Metabolism; Metabolism of Terpenoids and Polyketides; Biosynthesis of 12-, 14- and 16-

			membered macrolides
Biosynthesis of ansamycins	16947	19272	Metabolism; Metabolism of Terpenoids and Polyketides; Biosynthesis of ansamycins
Biosynthesis of siderophore group nonribosomal peptides	17390	21118	Metabolism; Metabolism of Terpenoids and Polyketides; Biosynthesis of siderophore group nonribosomal peptides
Biosynthesis of type II polyketide backbone	49	40	Metabolism; Metabolism of Terpenoids and Polyketides; Biosynthesis of type II polyketide backbone
Biosynthesis of type II polyketide products	69	154	Metabolism; Metabolism of Terpenoids and Polyketides; Biosynthesis of type II polyketide products
Biosynthesis of unsaturated fatty acids	97986	109450	Metabolism; Lipid Metabolism; Biosynthesis of unsaturated fatty acids
Biosynthesis of vancomycin group antibiotics	27800	28079	Metabolism; Metabolism of Terpenoids and Polyketides; Biosynthesis of vancomycin group antibiotics
Biotin metabolism	73925	77500	Metabolism; Metabolism of Cofactors and Vitamins; Biotin metabolism
Bisphenol degradation	21693	26936	Metabolism; Xenobiotics Biodegradation and Metabolism; Bisphenol degradation
Bladder cancer	1124	2007	Human Diseases; Cancers; Bladder cancer
Butanoate metabolism	255051	309712	Metabolism; Carbohydrate Metabolism; Butanoate metabolism
Butirosin and neomycin biosynthesis	27341	27057	Metabolism; Biosynthesis of Other Secondary Metabolites; Butirosin and neomycin biosynthesis
C5-Branched dibasic acid metabolism	114030	125446	Metabolism; Carbohydrate Metabolism; C5-Branched dibasic acid metabolism
Caffeine metabolism	385	574	Metabolism; Biosynthesis of Other Secondary Metabolites; Caffeine metabolism
Calcium signaling pathway	12081	10911	Environmental Information Processing; Signal Transduction; Calcium signaling pathway
Caprolactam degradation	36806	52758	Metabolism; Xenobiotics Biodegradation and Metabolism; Caprolactam degradation
Carbohydrate digestion and absorption	12851	12303	Organismal Systems; Digestive System; Carbohydrate digestion and absorption
Carbohydrate metabolism	109798	109195	Unclassified; Metabolism; Carbohydrate metabolism
Carbon fixation in photosynthetic organisms	322525	325851	Metabolism; Energy Metabolism; Carbon fixation in photosynthetic organisms
Carbon fixation pathways in prokaryotes	362069	403031	Metabolism; Energy Metabolism; Carbon fixation pathways in prokaryotes
Cardiac muscle contraction	11852	14842	Organismal Systems; Circulatory System; Cardiac muscle contraction
Carotenoid biosynthesis	101778	96282	Metabolism; Metabolism of Terpenoids and Polyketides; Carotenoid biosynthesis
Cell cycle	2	14	Cellular Processes; Cell Growth and Death; Cell cycle
Cell cycle - Caulobacter	259265	272113	Cellular Processes; Cell Growth and Death; Cell cycle - Caulobacter
Cell division	32979	36255	Unclassified; Cellular Processes and Signaling; Cell division
Cell motility and secretion	126034	133585	Unclassified; Cellular Processes and Signaling; Cell motility and secretion
Cellular antigens	31180	33796	Environmental Information Processing; Signaling Molecules and Interaction; Cellular antigens
Chagas disease (American trypanosomiasis)	4554	5839	Human Diseases; Infectious Diseases; Chagas disease (American trypanosomiasis)
Chaperones and folding catalysts	516036	540512	Genetic Information Processing; Folding, Sorting and Degradation; Chaperones and folding catalysts
Chloroalkane and chloroalkene degradation	108803	118155	Metabolism; Xenobiotics Biodegradation and Metabolism; Chloroalkane and chloroalkene degradation
Chlorocyclohexane and chlorobenzene degradation	46206	49629	Metabolism; Xenobiotics Biodegradation and Metabolism; Chlorocyclohexane and chlorobenzene degradation
Chromosome	698375	731287	Genetic Information Processing; Replication and Repair; Chromosome
Chronic myeloid leukemia	0	1	Human Diseases; Cancers; Chronic myeloid leukemia
Circadian rhythm - plant	1134	1814	Organismal Systems; Environmental

			Adaptation; Circadian rhythm - plant
Citrate cycle (TCA cycle)	245867	280071	Metabolism; Carbohydrate Metabolism; Citrate cycle (TCA cycle)
Clavulanic acid biosynthesis	96	36	Metabolism; Biosynthesis of Other Secondary Metabolites; Clavulanic acid biosynthesis
Colorectal cancer	5487	7226	Human Diseases; Cancers; Colorectal cancer
Cyanoamino acid metabolism	107593	114338	Metabolism; Metabolism of Other Amino Acids; Cyanoamino acid metabolism
Cysteine and methionine metabolism	330893	357210	Metabolism; Amino Acid Metabolism; Cysteine and methionine metabolism
Cytochrome P450	135	176	Metabolism; Enzyme Families; Cytochrome P450
Cytokine receptors	4	2	Environmental Information Processing; Signaling Molecules and Interaction; Cytokine receptors
Cytokine-cytokine receptor interaction	4	2	Environmental Information Processing; Signaling Molecules and Interaction; Cytokine-cytokine receptor interaction
Cytoskeleton proteins	162136	163234	Cellular Processes; Cell Motility; Cytoskeleton proteins
D-Alanine metabolism	31448	34837	Metabolism; Metabolism of Other Amino Acids; D-Alanine metabolism
D-Arginine and D-ornithine metabolism	633	994	Metabolism; Metabolism of Other Amino Acids; D-Arginine and D-ornithine metabolism
D-Glutamine and D-glutamate metabolism	63094	65869	Metabolism; Metabolism of Other Amino Acids; D-Glutamine and D-glutamate metabolism
DNA repair and recombination proteins	1182132	1243385	Genetic Information Processing; Replication and Repair; DNA repair and recombination proteins
DNA replication	233192	248222	Genetic Information Processing; Replication and Repair; DNA replication
DNA replication proteins	431171	456314	Genetic Information Processing; Replication and Repair; DNA replication proteins
Dilated cardiomyopathy (DCM)	1	0	Human Diseases; Cardiovascular Diseases; Dilated cardiomyopathy (DCM)
Dioxin degradation	31555	33848	Metabolism; Xenobiotics Biodegradation and Metabolism; Dioxin degradation
Drug metabolism - cytochrome P450	121418	129405	Metabolism; Xenobiotics Biodegradation and Metabolism; Drug metabolism - cytochrome P450
Drug metabolism - other enzymes	55862	66221	Metabolism; Xenobiotics Biodegradation and Metabolism; Drug metabolism - other enzymes
Electron transfer carriers	14958	16358	Unclassified; Cellular Processes and Signaling; Electron transfer carriers
Endocrine and other factor-regulated calcium reabsorption	1	0	Organismal Systems; Excretory System; Endocrine and other factor-regulated calcium reabsorption
Endocytosis	37	91	Cellular Processes; Transport and Catabolism; Endocytosis
Energy metabolism	259355	296283	Unclassified; Metabolism; Energy metabolism
Epithelial cell signaling in Helicobacter pylori infection	26963	27590	Human Diseases; Infectious Diseases; Epithelial cell signaling in Helicobacter pylori infection
Ether lipid metabolism	821	1627	Metabolism; Lipid Metabolism; Ether lipid metabolism
Ethylbenzene degradation	19720	23562	Metabolism; Xenobiotics Biodegradation and Metabolism; Ethylbenzene degradation
Fatty acid biosynthesis	215163	232007	Metabolism; Lipid Metabolism; Fatty acid biosynthesis
Fatty acid elongation in mitochondria	1	2	Metabolism; Lipid Metabolism; Fatty acid elongation in mitochondria
Fatty acid metabolism	215786	250382	Metabolism; Lipid Metabolism; Fatty acid metabolism
Fc gamma R-mediated phagocytosis	37	91	Organismal Systems; Immune System; Fc gamma R-mediated phagocytosis
Flagellar assembly	61259	115477	Cellular Processes; Cell Motility; Flagellar assembly
Flavone and flavonol biosynthesis	43	135	Metabolism; Biosynthesis of Other Secondary Metabolites; Flavone and flavonol biosynthesis
Flavonoid biosynthesis	37907	35454	Metabolism; Biosynthesis of Other Secondary Metabolites; Flavonoid biosynthesis
Fluorobenzoate degradation	31217	34316	Metabolism; Xenobiotics Biodegradation and Metabolism; Fluorobenzoate degradation

Focal adhesion	1	0	Cellular Processes; Cell Communication; Focal adhesion
Folate biosynthesis	210189	222194	Metabolism; Metabolism of Cofactors and Vitamins; Folate biosynthesis
Fructose and mannose metabolism	305554	315602	Metabolism; Carbohydrate Metabolism; Fructose and mannose metabolism
Function unknown	637236	714153	Unclassified; Poorly Characterized; Function unknown
G protein-coupled receptors	17	76	Environmental Information Processing; Signaling Molecules and Interaction; G protein-coupled receptors
Galactose metabolism	142191	154442	Metabolism; Carbohydrate Metabolism; Galactose metabolism
Gastric acid secretion	1	0	Organismal Systems; Digestive System; Gastric acid secretion
General function prediction only	1704553	1799146	Unclassified; Poorly Characterized; General function prediction only
Geraniol degradation	56459	76947	Metabolism; Metabolism of Terpenoids and Polyketides; Geraniol degradation
Germination	2058	2027	Unclassified; Cellular Processes and Signaling; Germination
Glutamatergic synapse	45635	46469	Organismal Systems; Nervous System; Glutamatergic synapse
Glutathione metabolism	235481	255269	Metabolism; Metabolism of Other Amino Acids; Glutathione metabolism
Glycan biosynthesis and metabolism	29437	32778	Unclassified; Metabolism; Glycan biosynthesis and metabolism
Glycerolipid metabolism	150548	157894	Metabolism; Lipid Metabolism; Glycerolipid metabolism
Glycerophospholipid metabolism	195401	216014	Metabolism; Lipid Metabolism; Glycerophospholipid metabolism
Glycine, serine and threonine metabolism	313882	349916	Metabolism; Amino Acid Metabolism; Glycine, serine and threonine metabolism
Glycolysis / Gluconeogenesis	504855	528161	Metabolism; Carbohydrate Metabolism; Glycolysis / Gluconeogenesis
Glycosaminoglycan degradation	3080	5295	Metabolism; Glycan Biosynthesis and Metabolism; Glycosaminoglycan degradation
Glycosphingolipid biosynthesis - ganglio series	1990	3416	Metabolism; Glycan Biosynthesis and Metabolism; Glycosphingolipid biosynthesis - ganglio series
Glycosphingolipid biosynthesis - globo series	3648	5924	Metabolism; Glycan Biosynthesis and Metabolism; Glycosphingolipid biosynthesis - globo series
Glycosphingolipid biosynthesis - lacto and neolacto series	31	98	Metabolism; Glycan Biosynthesis and Metabolism; Glycosphingolipid biosynthesis - lacto and neolacto series
Glycosylphosphatidylinositol(GPI)-anchor biosynthesis	3	0	Metabolism; Glycan Biosynthesis and Metabolism; Glycosylphosphatidylinositol(GPI)-anchor biosynthesis
Glycosyltransferases	260557	261461	Metabolism; Glycan Biosynthesis and Metabolism; Glycosyltransferases
Glyoxylate and dicarboxylate metabolism	250193	285768	Metabolism; Carbohydrate Metabolism; Glyoxylate and dicarboxylate metabolism
GnRH signaling pathway	37	91	Organismal Systems; Endocrine System; GnRH signaling pathway
Hematopoietic cell lineage	8	12	Organismal Systems; Immune System; Hematopoietic cell lineage
Hepatitis C	2	14	Human Diseases; Infectious Diseases; Hepatitis C
Histidine metabolism	212404	229028	Metabolism; Amino Acid Metabolism; Histidine metabolism
Homologous recombination	354149	371445	Genetic Information Processing; Replication and Repair; Homologous recombination
Huntington's disease	48620	55078	Human Diseases; Neurodegenerative Diseases; Huntington's disease
Hypertrophic cardiomyopathy (HCM)	178	261	Human Diseases; Cardiovascular Diseases; Hypertrophic cardiomyopathy (HCM)
Indole alkaloid biosynthesis	57	189	Metabolism; Biosynthesis of Other Secondary Metabolites; Indole alkaloid biosynthesis
Influenza A	5493	7245	Human Diseases; Infectious Diseases; Influenza A
Inorganic ion transport and metabolism	81293	102839	Unclassified; Cellular Processes and Signaling; Inorganic ion transport and metabolism
Inositol phosphate metabolism	59633	68616	Metabolism; Carbohydrate Metabolism;

			Inositol phosphate metabolism
Insulin signaling pathway	76655	72766	Organismal Systems; Endocrine System; Insulin signaling pathway
Ion channels	14475	15014	Environmental Information Processing; Signaling Molecules and Interaction; Ion channels
Isoflavonoid biosynthesis	24	41	Metabolism; Biosynthesis of Other Secondary Metabolites; Isoflavonoid biosynthesis
Isoquinoline alkaloid biosynthesis	19648	22116	Metabolism; Biosynthesis of Other Secondary Metabolites; Isoquinoline alkaloid biosynthesis
Leishmaniasis	0	2	Human Diseases; Infectious Diseases; Leishmaniasis
Leukocyte transendothelial migration	1	0	Organismal Systems; Immune System; Leukocyte transendothelial migration
Limonene and pinene degradation	69031	88626	Metabolism; Metabolism of Terpenoids and Polyketides; Limonene and pinene degradation
Linoleic acid metabolism	5268	8810	Metabolism; Lipid Metabolism; Linoleic acid metabolism
Lipid biosynthesis proteins	310742	334751	Metabolism; Lipid Metabolism; Lipid biosynthesis proteins
Lipid metabolism	75918	79376	Unclassified; Metabolism; Lipid metabolism
Lipoic acid metabolism	58807	58371	Metabolism; Metabolism of Cofactors and Vitamins; Lipoic acid metabolism
Lipopolysaccharide biosynthesis	101377	113232	Metabolism; Glycan Biosynthesis and Metabolism; Lipopolysaccharide biosynthesis
Lipopolysaccharide biosynthesis proteins	122844	143524	Metabolism; Glycan Biosynthesis and Metabolism; Lipopolysaccharide biosynthesis proteins
Lysine biosynthesis	255583	275065	Metabolism; Amino Acid Metabolism; Lysine biosynthesis
Lysine degradation	97137	123725	Metabolism; Amino Acid Metabolism; Lysine degradation
Lysosome	4602	7896	Cellular Processes; Transport and Catabolism; Lysosome
MAPK signaling pathway - yeast	16082	18028	Environmental Information Processing; Signal Transduction; MAPK signaling pathway - yeast
Measles	2	14	Human Diseases; Infectious Diseases; Measles
Meiosis - yeast	51606	48271	Cellular Processes; Cell Growth and Death; Meiosis - yeast
Melanogenesis	44	114	Organismal Systems; Endocrine System; Melanogenesis
Membrane and intracellular structural molecules	263325	301272	Unclassified; Cellular Processes and Signaling; Membrane and intracellular structural molecules
Metabolism of cofactors and vitamins	77260	84987	Unclassified; Metabolism; Metabolism of cofactors and vitamins
Metabolism of xenobiotics by cytochrome P450	120562	128466	Metabolism; Xenobiotics Biodegradation and Metabolism; Metabolism of xenobiotics by cytochrome P450
Methane metabolism	445565	468285	Metabolism; Energy Metabolism; Methane metabolism
Mineral absorption	37521	35150	Organismal Systems; Digestive System; Mineral absorption
Mismatch repair	319428	333079	Genetic Information Processing; Replication and Repair; Mismatch repair
N-Glycan biosynthesis	38945	36793	Metabolism; Glycan Biosynthesis and Metabolism; N-Glycan biosynthesis
NOD-like receptor signaling pathway	15975	16038	Organismal Systems; Immune System; NOD-like receptor signaling pathway
Naphthalene degradation	82261	91047	Metabolism; Xenobiotics Biodegradation and Metabolism; Naphthalene degradation
Neuroactive ligand-receptor interaction	3	5	Environmental Information Processing; Signaling Molecules and Interaction; Neuroactive ligand-receptor interaction
Nicotinate and nicotinamide metabolism	182385	196210	Metabolism; Metabolism of Cofactors and Vitamins; Nicotinate and nicotinamide metabolism
Nitrogen metabolism	269172	297689	Metabolism; Energy Metabolism; Nitrogen metabolism
Nitrotoluene degradation	19305	22781	Metabolism; Xenobiotics Biodegradation and Metabolism; Nitrotoluene degradation
Non-homologous end-joining	5512	7762	Genetic Information Processing; Replication and Repair; Non-homologous end-joining

Notch signaling pathway	0	1	Environmental Information Processing; Signal Transduction; Notch signaling pathway
Novobiocin biosynthesis	48917	53558	Metabolism; Biosynthesis of Other Secondary Metabolites; Novobiocin biosynthesis
Nucleotide excision repair	137327	144380	Genetic Information Processing; Replication and Repair; Nucleotide excision repair
Nucleotide metabolism	3651	7068	Unclassified; Metabolism; Nucleotide metabolism
One carbon pool by folate	213824	228369	Metabolism; Metabolism of Cofactors and Vitamins; One carbon pool by folate
Other glycan degradation	21760	27428	Metabolism; Glycan Biosynthesis and Metabolism; Other glycan degradation
Other ion-coupled transporters	413842	474044	Unclassified; Cellular Processes and Signaling; Other ion-coupled transporters
Other transporters	122020	132201	Unclassified; Cellular Processes and Signaling; Other transporters
Others	575581	591455	Unclassified; Metabolism; Others
Oxidative phosphorylation	725064	754474	Metabolism; Energy Metabolism; Oxidative phosphorylation
PPAR signaling pathway	77190	82356	Organismal Systems; Endocrine System; PPAR signaling pathway
Pancreatic secretion	4	5	Organismal Systems; Digestive System; Pancreatic secretion
Pantothenate and CoA biosynthesis	268345	281208	Metabolism; Metabolism of Cofactors and Vitamins; Pantothenate and CoA biosynthesis
Parkinson's disease	17342	22081	Human Diseases; Neurodegenerative Diseases; Parkinson's disease
Pathogenic Escherichia coli infection	1	0	Human Diseases; Infectious Diseases; Pathogenic Escherichia coli infection
Pathways in cancer	38475	41009	Human Diseases; Cancers; Pathways in cancer
Penicillin and cephalosporin biosynthesis	16641	19351	Metabolism; Biosynthesis of Other Secondary Metabolites; Penicillin and cephalosporin biosynthesis
Pentose and glucuronate interconversions	122994	140385	Metabolism; Carbohydrate Metabolism; Pentose and glucuronate interconversions
Pentose phosphate pathway	351792	368325	Metabolism; Carbohydrate Metabolism; Pentose phosphate pathway
Peptidases	919137	938869	Metabolism; Enzyme Families; Peptidases
Peptidoglycan biosynthesis	312455	328482	Metabolism; Glycan Biosynthesis and Metabolism; Peptidoglycan biosynthesis
Peroxisome	100759	110823	Cellular Processes; Transport and Catabolism; Peroxisome
Pertussis	17714	24045	Human Diseases; Infectious Diseases; Pertussis
Phagosome	3	0	Cellular Processes; Transport and Catabolism; Phagosome
Phenylalanine metabolism	110654	127668	Metabolism; Amino Acid Metabolism; Phenylalanine metabolism
Phenylalanine, tyrosine and tryptophan biosynthesis	308740	327511	Metabolism; Amino Acid Metabolism; Phenylalanine, tyrosine and tryptophan biosynthesis
Phenylpropanoid biosynthesis	58161	59994	Metabolism; Biosynthesis of Other Secondary Metabolites; Phenylpropanoid biosynthesis
Phosphatidylinositol signaling system	61448	62669	Environmental Information Processing; Signal Transduction; Phosphatidylinositol signaling system
Phosphonate and phosphinate metabolism	5351	10197	Metabolism; Metabolism of Other Amino Acids; Phosphonate and phosphinate metabolism
Phosphotransferase system (PTS)	26487	49888	Environmental Information Processing; Membrane Transport; Phosphotransferase system (PTS)
Photosynthesis	851821	789116	Metabolism; Energy Metabolism; Photosynthesis
Photosynthesis - antenna proteins	205372	185490	Metabolism; Energy Metabolism; Photosynthesis - antenna proteins
Photosynthesis proteins	1071846	990230	Metabolism; Energy Metabolism; Photosynthesis proteins
Phototransduction - fly	1	0	Organismal Systems; Sensory System; Phototransduction - fly
Plant-pathogen interaction	39250	44630	Organismal Systems; Environmental Adaptation; Plant-pathogen interaction
Polycyclic aromatic hydrocarbon degradation	59562	60786	Metabolism; Xenobiotics Biodegradation and Metabolism; Polycyclic aromatic hydrocarbon

			degradation
Polyketide sugar unit biosynthesis	95262	95422	Metabolism; Metabolism of Terpenoids and Polyketides; Polyketide sugar unit biosynthesis
Pores ion channels	282112	309621	Unclassified; Cellular Processes and Signaling; Pores ion channels
Porphyrin and chlorophyll metabolism	828519	812821	Metabolism; Metabolism of Cofactors and Vitamins; Porphyrin and chlorophyll metabolism
Prenyltransferases	232592	231116	Metabolism; Metabolism of Terpenoids and Polyketides; Prenyltransferases
Primary bile acid biosynthesis	1274	2250	Metabolism; Lipid Metabolism; Primary bile acid biosynthesis
Primary immunodeficiency	16050	17843	Human Diseases; Immune System Diseases; Primary immunodeficiency
Prion diseases	1458	2848	Human Diseases; Neurodegenerative Diseases; Prion diseases
Progesterone-mediated oocyte maturation	15963	16020	Organismal Systems; Endocrine System; Progesterone-mediated oocyte maturation
Propanoate metabolism	226268	273714	Metabolism; Carbohydrate Metabolism; Propanoate metabolism
Prostate cancer	15965	16024	Human Diseases; Cancers; Prostate cancer
Proteasome	16768	16761	Genetic Information Processing; Folding, Sorting and Degradation; Proteasome
Protein digestion and absorption	1008	1800	Organismal Systems; Digestive System; Protein digestion and absorption
Protein export	261189	274355	Genetic Information Processing; Folding, Sorting and Degradation; Protein export
Protein folding and associated processing	437842	452927	Unclassified; Genetic Information Processing; Protein folding and associated processing
Protein kinases	291294	294816	Metabolism; Enzyme Families; Protein kinases
Protein processing in endoplasmic reticulum	54697	52933	Genetic Information Processing; Folding, Sorting and Degradation; Protein processing in endoplasmic reticulum
Proximal tubule bicarbonate reclamation	14457	15078	Organismal Systems; Excretory System; Proximal tubule bicarbonate reclamation
Purine metabolism	920757	976993	Metabolism; Nucleotide Metabolism; Purine metabolism
Pyrimidine metabolism	616354	653968	Metabolism; Nucleotide Metabolism; Pyrimidine metabolism
Pyruvate metabolism	488030	521995	Metabolism; Carbohydrate Metabolism; Pyruvate metabolism
RIG-I-like receptor signaling pathway	720	765	Organismal Systems; Immune System; RIG-I-like receptor signaling pathway
RNA degradation	268342	272844	Genetic Information Processing; Folding, Sorting and Degradation; RNA degradation
RNA polymerase	80795	82363	Genetic Information Processing; Transcription; RNA polymerase
RNA transport	40236	41423	Genetic Information Processing; Translation; RNA transport
Regulation of actin cytoskeleton	1	0	Cellular Processes; Cell Motility; Regulation of actin cytoskeleton
Renal cell carcinoma	17025	17760	Human Diseases; Cancers; Renal cell carcinoma
Renin-angiotensin system	469	668	Organismal Systems; Endocrine System; Renin-angiotensin system
Replication, recombination and repair proteins	443521	452286	Unclassified; Genetic Information Processing; Replication, recombination and repair proteins
Restriction enzyme	53754	54005	Unclassified; Genetic Information Processing; Restriction enzyme
Retinol metabolism	56469	57681	Metabolism; Metabolism of Cofactors and Vitamins; Retinol metabolism
Riboflavin metabolism	149261	155634	Metabolism; Metabolism of Cofactors and Vitamins; Riboflavin metabolism
Ribosome	935870	972158	Genetic Information Processing; Translation; Ribosome
Ribosome Biogenesis	502139	541499	Genetic Information Processing; Translation; Ribosome Biogenesis
Ribosome biogenesis in eukaryotes	18474	20461	Genetic Information Processing; Translation; Ribosome biogenesis in eukaryotes
Salivary secretion	1	0	Organismal Systems; Digestive System; Salivary secretion
Secondary bile acid biosynthesis	385	636	Metabolism; Lipid Metabolism; Secondary bile acid biosynthesis

Secretion system	737696	826902	Environmental Information Processing; Membrane Transport; Secretion system
Selenocompound metabolism	145993	158133	Metabolism; Metabolism of Other Amino Acids; Selenocompound metabolism
Sesquiterpenoid biosynthesis	10	17	Metabolism; Metabolism of Terpenoids and Polyketides; Sesquiterpenoid biosynthesis
Shigellosis	116	442	Human Diseases; Infectious Diseases; Shigellosis
Signal transduction mechanisms	271634	282453	Unclassified; Cellular Processes and Signaling; Signal transduction mechanisms
Small cell lung cancer	5487	7228	Human Diseases; Cancers; Small cell lung cancer
Sphingolipid metabolism	18584	21506	Metabolism; Lipid Metabolism; Sphingolipid metabolism
Sporulation	34020	34857	Unclassified; Cellular Processes and Signaling; Sporulation
Staphylococcus aureus infection	1571	2666	Human Diseases; Infectious Diseases; Staphylococcus aureus infection
Starch and sucrose metabolism	367878	374326	Metabolism; Carbohydrate Metabolism; Starch and sucrose metabolism
Steroid biosynthesis	24821	22846	Metabolism; Lipid Metabolism; Steroid biosynthesis
Steroid hormone biosynthesis	14549	14687	Metabolism; Lipid Metabolism; Steroid hormone biosynthesis
Stilbenoid, diarylheptanoid and gingerol biosynthesis	26236	24959	Metabolism; Biosynthesis of Other Secondary Metabolites; Stilbenoid, diarylheptanoid and gingerol biosynthesis
Streptomycin biosynthesis	169510	171012	Metabolism; Biosynthesis of Other Secondary Metabolites; Streptomycin biosynthesis
Styrene degradation	22942	29875	Metabolism; Xenobiotics Biodegradation and Metabolism; Styrene degradation
Sulfur metabolism	149504	160077	Metabolism; Energy Metabolism; Sulfur metabolism
Sulfur relay system	188365	195701	Genetic Information Processing; Folding, Sorting and Degradation; Sulfur relay system
Synthesis and degradation of ketone bodies	15448	26137	Metabolism; Lipid Metabolism; Synthesis and degradation of ketone bodies
Systemic lupus erythematosus	57	87	Human Diseases; Immune System Diseases; Systemic lupus erythematosus
Taurine and hypotaurine metabolism	47526	52564	Metabolism; Metabolism of Other Amino Acids; Taurine and hypotaurine metabolism
Terpenoid backbone biosynthesis	240308	250720	Metabolism; Metabolism of Terpenoids and Polyketides; Terpenoid backbone biosynthesis
Tetracycline biosynthesis	71195	75070	Metabolism; Metabolism of Terpenoids and Polyketides; Tetracycline biosynthesis
Thiamine metabolism	176800	186116	Metabolism; Metabolism of Cofactors and Vitamins; Thiamine metabolism
Tight junction	1	0	Cellular Processes; Cell Communication; Tight junction
Toluene degradation	77258	85826	Metabolism; Xenobiotics Biodegradation and Metabolism; Toluene degradation
Toxoplasmosis	5487	7226	Human Diseases; Infectious Diseases; Toxoplasmosis
Transcription factors	445041	521814	Genetic Information Processing; Transcription; Transcription factors
Transcription machinery	331412	351342	Genetic Information Processing; Transcription; Transcription machinery
Transcription related proteins	1135	2337	Unclassified; Genetic Information Processing; Transcription related proteins
Translation factors	212820	221028	Genetic Information Processing; Translation; Translation factors
Translation proteins	387469	406983	Unclassified; Genetic Information Processing; Translation proteins
Transporters	2514549	2648494	Environmental Information Processing; Membrane Transport; Transporters
Tropane, piperidine and pyridine alkaloid biosynthesis	49491	54244	Metabolism; Biosynthesis of Other Secondary Metabolites; Tropane, piperidine and pyridine alkaloid biosynthesis
Tryptophan metabolism	133934	166869	Metabolism; Amino Acid Metabolism; Tryptophan metabolism
Tuberculosis	152222	147306	Human Diseases; Infectious Diseases; Tuberculosis
Two-component system	931021	1023531	Environmental Information Processing; Signal Transduction; Two-component system
Type I diabetes mellitus	29963	29675	Human Diseases; Metabolic Diseases; Type I

			diabetes mellitus
Type II diabetes mellitus	39124	38047	Human Diseases; Metabolic Diseases; Type II diabetes mellitus
Tyrosine metabolism	188105	203990	Metabolism; Amino Acid Metabolism; Tyrosine metabolism
Ubiquinone and other terpenoid-quinone biosynthesis	290082	289640	Metabolism; Metabolism of Cofactors and Vitamins; Ubiquinone and other terpenoid-quinone biosynthesis
Ubiquitin system	13176	12794	Genetic Information Processing; Folding, Sorting and Degradation; Ubiquitin system
VEGF signaling pathway	0	2	Environmental Information Processing; Signal Transduction; VEGF signaling pathway
Valine, leucine and isoleucine biosynthesis	326067	346197	Metabolism; Amino Acid Metabolism; Valine, leucine and isoleucine biosynthesis
Valine, leucine and isoleucine degradation	193648	241348	Metabolism; Amino Acid Metabolism; Valine, leucine and isoleucine degradation
Various types of N-glycan biosynthesis	7	17	Metabolism; Glycan Biosynthesis and Metabolism; Various types of N-glycan biosynthesis
Vasopressin-regulated water reabsorption	4	4	Organismal Systems; Excretory System; Vasopressin-regulated water reabsorption
Vibrio cholerae infection	109	184	Human Diseases; Infectious Diseases; Vibrio cholerae infection
Vibrio cholerae pathogenic cycle	67634	68721	Human Diseases; Infectious Diseases; Vibrio cholerae pathogenic cycle
Viral myocarditis	5488	7226	Human Diseases; Cardiovascular Diseases; Viral myocarditis
Vitamin B6 metabolism	75547	81753	Metabolism; Metabolism of Cofactors and Vitamins; Vitamin B6 metabolism
Wnt signaling pathway	0	1	Environmental Information Processing; Signal Transduction; Wnt signaling pathway
Xylene degradation	29865	30432	Metabolism; Xenobiotics Biodegradation and Metabolism; Xylene degradation
Zeatin biosynthesis	17273	17976	Metabolism; Metabolism of Terpenoids and Polyketides; Zeatin biosynthesis
alpha-Linolenic acid metabolism	15753	17722	Metabolism; Lipid Metabolism; alpha-Linolenic acid metabolism
beta-Alanine metabolism	126329	148008	Metabolism; Metabolism of Other Amino Acids; beta-Alanine metabolism
beta-Lactam resistance	15451	17358	Metabolism; Biosynthesis of Other Secondary Metabolites; beta-Lactam resistance
mRNA surveillance pathway	17	43	Genetic Information Processing; Translation; mRNA surveillance pathway
mTOR signaling pathway	2	14	Environmental Information Processing; Signal Transduction; mTOR signaling pathway
p53 signaling pathway	5542	7320	Cellular Processes; Cell Growth and Death; p53 signaling pathway

Table S4 Results of PICRUSt at level 3 KEGG Orthology using the V5-V7 amplification regions

OTU ID	A1(V5V7)	B1(V5V7)	KEGG Pathways
1,1,1-Trichloro-2,2-bis(4-chlorophenyl)ethane (DDT) degradation	93	72	Metabolism; Xenobiotics Biodegradation and Metabolism; 1,1,1-Trichloro-2,2-bis(4-chlorophenyl)ethane (DDT) degradation
ABC transporters	1152756	1034806	Environmental Information Processing; Membrane Transport; ABC transporters
Adherens junction	2	0	Cellular Processes; Cell Communication; Adherens junction
Adipocytokine signaling pathway	23664	19686	Organismal Systems; Endocrine System; Adipocytokine signaling pathway
African trypanosomiasis	7975	7534	Human Diseases; Infectious Diseases; African trypanosomiasis
Alanine, aspartate and glutamate metabolism	259550	244164	Metabolism; Amino Acid Metabolism; Alanine, aspartate and glutamate metabolism
Alzheimer's disease	36604	36688	Human Diseases; Neurodegenerative Diseases; Alzheimer's disease
Amino acid metabolism	66801	66012	Unclassified; Metabolism; Amino acid metabolism
Amino acid related enzymes	345277	338639	Metabolism; Amino Acid Metabolism; Amino acid related enzymes
Amino sugar and nucleotide sugar metabolism	303066	302779	Metabolism; Carbohydrate Metabolism; Amino sugar and nucleotide sugar metabolism
Aminoacyl-tRNA biosynthesis	251361	237375	Genetic Information Processing; Translation; Aminoacyl-tRNA biosynthesis
Aminobenzoate degradation	143078	124027	Metabolism; Xenobiotics Biodegradation and Metabolism; Aminobenzoate degradation
Amoebiasis	3426	2467	Human Diseases; Infectious Diseases; Amoebiasis
Amyotrophic lateral sclerosis (ALS)	24767	25365	Human Diseases; Neurodegenerative Diseases; Amyotrophic lateral sclerosis (ALS)
Antigen processing and presentation	4409	4298	Organismal Systems; Immune System; Antigen processing and presentation
Apoptosis	11190	11129	Cellular Processes; Cell Growth and Death; Apoptosis
Arachidonic acid metabolism	25529	26092	Metabolism; Lipid Metabolism; Arachidonic acid metabolism
Arginine and proline metabolism	375828	359626	Metabolism; Amino Acid Metabolism; Arginine and proline metabolism
Arrhythmogenic right ventricular cardiomyopathy (ARVC)	2	0	Human Diseases; Cardiovascular Diseases; Arrhythmogenic right ventricular cardiomyopathy (ARVC)
Ascorbate and aldarate metabolism	73054	76515	Metabolism; Carbohydrate Metabolism; Ascorbate and aldarate metabolism
Atrazine degradation	19057	16905	Metabolism; Xenobiotics Biodegradation and Metabolism; Atrazine degradation
Bacterial chemotaxis	215416	218047	Cellular Processes; Cell Motility; Bacterial chemotaxis
Bacterial invasion of epithelial cells	365	508	Human Diseases; Infectious Diseases; Bacterial invasion of epithelial cells
Bacterial motility proteins	519699	530613	Cellular Processes; Cell Motility; Bacterial motility proteins
Bacterial secretion system	267338	287188	Environmental Information Processing; Membrane Transport; Bacterial secretion system
Bacterial toxins	22578	20863	Environmental Information Processing; Signaling Molecules and Interaction; Bacterial toxins
Basal transcription factors	1527	962	Genetic Information Processing; Transcription; Basal transcription factors
Base excision repair	133749	122252	Genetic Information Processing; Replication and Repair; Base excision repair
Benzoate degradation	197968	181667	Metabolism; Xenobiotics Biodegradation and Metabolism; Benzoate degradation
Betalain biosynthesis	286	260	Metabolism; Biosynthesis of Other Secondary Metabolites; Betalain biosynthesis
Bile secretion	933	1086	Organismal Systems; Digestive System; Bile secretion
Biosynthesis and biodegradation of secondary metabolites	36064	39319	Unclassified; Metabolism; Biosynthesis and biodegradation of secondary metabolites
Biosynthesis of 12-, 14- and 16-membered macrolides	64	30	Metabolism; Metabolism of Terpenoids and Polyketides; Biosynthesis of 12-, 14- and 16-membered macrolides

Biosynthesis of ansamycins	19017	20356	Metabolism; Metabolism of Terpenoids and Polyketides; Biosynthesis of ansamycins
Biosynthesis of siderophore group nonribosomal peptides	23142	26699	Metabolism; Metabolism of Terpenoids and Polyketides; Biosynthesis of siderophore group nonribosomal peptides
Biosynthesis of type II polyketide backbone	136	36	Metabolism; Metabolism of Terpenoids and Polyketides; Biosynthesis of type II polyketide backbone
Biosynthesis of type II polyketide products	202	177	Metabolism; Metabolism of Terpenoids and Polyketides; Biosynthesis of type II polyketide products
Biosynthesis of unsaturated fatty acids	84219	79866	Metabolism; Lipid Metabolism; Biosynthesis of unsaturated fatty acids
Biosynthesis of vancomycin group antibiotics	12205	11816	Metabolism; Metabolism of Terpenoids and Polyketides; Biosynthesis of vancomycin group antibiotics
Biotin metabolism	39729	41719	Metabolism; Metabolism of Cofactors and Vitamins; Biotin metabolism
Bisphenol degradation	34217	23824	Metabolism; Xenobiotics Biodegradation and Metabolism; Bisphenol degradation
Bladder cancer	5210	5289	Human Diseases; Cancers; Bladder cancer
Butanoate metabolism	352235	330090	Metabolism; Carbohydrate Metabolism; Butanoate metabolism
Butirosin and neomycin biosynthesis	11887	10534	Metabolism; Biosynthesis of Other Secondary Metabolites; Butirosin and neomycin biosynthesis
C5-Branched dibasic acid metabolism	89214	89350	Metabolism; Carbohydrate Metabolism; C5-Branched dibasic acid metabolism
CAM ligands	1	0	Environmental Information Processing; Signaling Molecules and Interaction; CAM ligands
Caffeine metabolism	1404	977	Metabolism; Biosynthesis of Other Secondary Metabolites; Caffeine metabolism
Calcium signaling pathway	6	8	Environmental Information Processing; Signal Transduction; Calcium signaling pathway
Caprolactam degradation	95406	88503	Metabolism; Xenobiotics Biodegradation and Metabolism; Caprolactam degradation
Carbohydrate digestion and absorption	3715	4075	Organismal Systems; Digestive System; Carbohydrate digestion and absorption
Carbohydrate metabolism	52976	53374	Unclassified; Metabolism; Carbohydrate metabolism
Carbon fixation in photosynthetic organisms	142109	143737	Metabolism; Energy Metabolism; Carbon fixation in photosynthetic organisms
Carbon fixation pathways in prokaryotes	303653	297220	Metabolism; Energy Metabolism; Carbon fixation pathways in prokaryotes
Cardiac muscle contraction	13684	13629	Organismal Systems; Circulatory System; Cardiac muscle contraction
Carotenoid biosynthesis	15968	16003	Metabolism; Metabolism of Terpenoids and Polyketides; Carotenoid biosynthesis
Cell cycle - Caulobacter	158251	155004	Cellular Processes; Cell Growth and Death; Cell cycle - Caulobacter
Cell division	22925	24507	Unclassified; Cellular Processes and Signaling; Cell division
Cell motility and secretion	69534	73655	Unclassified; Cellular Processes and Signaling; Cell motility and secretion
Cellular antigens	24577	24363	Environmental Information Processing; Signaling Molecules and Interaction; Cellular antigens
Chagas disease (American trypanosomiasis)	7384	7580	Human Diseases; Infectious Diseases; Chagas disease (American trypanosomiasis)
Chaperones and folding catalysts	266529	277970	Genetic Information Processing; Folding, Sorting and Degradation; Chaperones and folding catalysts
Chloroalkane and chloroalkene degradation	89540	80254	Metabolism; Xenobiotics Biodegradation and Metabolism; Chloroalkane and chloroalkene degradation
Chlorocyclohexane and chlorobenzene degradation	35189	31807	Metabolism; Xenobiotics Biodegradation and Metabolism; Chlorocyclohexane and chlorobenzene degradation
Chromosome	376076	378205	Genetic Information Processing; Replication and Repair; Chromosome
Chronic myeloid leukemia	26	2	Human Diseases; Cancers; Chronic myeloid leukemia
Circadian rhythm - plant	3534	3096	Organismal Systems; Environmental Adaptation; Circadian rhythm - plant

Citrate cycle (TCA cycle)	236595	226898	Metabolism; Carbohydrate Metabolism; Citrate cycle (TCA cycle)
Clavulanic acid biosynthesis	308	32	Metabolism; Biosynthesis of Other Secondary Metabolites; Clavulanic acid biosynthesis
Colorectal cancer	10840	10854	Human Diseases; Cancers; Colorectal cancer
Cyanoamino acid metabolism	76097	76413	Metabolism; Metabolism of Other Amino Acids; Cyanoamino acid metabolism
Cysteine and methionine metabolism	247723	246344	Metabolism; Amino Acid Metabolism; Cysteine and methionine metabolism
Cytochrome P450	512	686	Metabolism; Enzyme Families; Cytochrome P450
Cytokine receptors	8	2	Environmental Information Processing; Signaling Molecules and Interaction; Cytokine receptors
Cytokine-cytokine receptor interaction	8	2	Environmental Information Processing; Signaling Molecules and Interaction; Cytokine-cytokine receptor interaction
Cytoskeleton proteins	63833	61688	Cellular Processes; Cell Motility; Cytoskeleton proteins
D-Alanine metabolism	26835	24981	Metabolism; Metabolism of Other Amino Acids; D-Alanine metabolism
D-Arginine and D-ornithine metabolism	2610	2027	Metabolism; Metabolism of Other Amino Acids; D-Arginine and D-ornithine metabolism
D-Glutamine and D-glutamate metabolism	33240	32994	Metabolism; Metabolism of Other Amino Acids; D-Glutamine and D-glutamate metabolism
DNA repair and recombination proteins	682319	659607	Genetic Information Processing; Replication and Repair; DNA repair and recombination proteins
DNA replication	139337	135039	Genetic Information Processing; Replication and Repair; DNA replication
DNA replication proteins	245526	242881	Genetic Information Processing; Replication and Repair; DNA replication proteins
Dilated cardiomyopathy (DCM)	2	0	Human Diseases; Cardiovascular Diseases; Dilated cardiomyopathy (DCM)
Dioxin degradation	28748	29829	Metabolism; Xenobiotics Biodegradation and Metabolism; Dioxin degradation
Drug metabolism - cytochrome P450	83248	80680	Metabolism; Xenobiotics Biodegradation and Metabolism; Drug metabolism - cytochrome P450
Drug metabolism - other enzymes	71068	67244	Metabolism; Xenobiotics Biodegradation and Metabolism; Drug metabolism - other enzymes
ECM-receptor interaction	1	0	Environmental Information Processing; Signaling Molecules and Interaction; ECM-receptor interaction
Electron transfer carriers	14876	16086	Unclassified; Cellular Processes and Signaling; Electron transfer carriers
Endocytosis	14	8	Cellular Processes; Transport and Catabolism; Endocytosis
Energy metabolism	240142	241964	Unclassified; Metabolism; Energy metabolism
Epithelial cell signaling in Helicobacter pylori infection	11296	12120	Human Diseases; Infectious Diseases; Epithelial cell signaling in Helicobacter pylori infection
Ether lipid metabolism	2912	2684	Metabolism; Lipid Metabolism; Ether lipid metabolism
Ethylbenzene degradation	27546	23673	Metabolism; Xenobiotics Biodegradation and Metabolism; Ethylbenzene degradation
Fatty acid biosynthesis	151166	142432	Metabolism; Lipid Metabolism; Fatty acid biosynthesis
Fatty acid elongation in mitochondria	6	15	Metabolism; Lipid Metabolism; Fatty acid elongation in mitochondria
Fatty acid metabolism	255550	234546	Metabolism; Lipid Metabolism; Fatty acid metabolism
Fc gamma R-mediated phagocytosis	14	8	Organismal Systems; Immune System; Fc gamma R-mediated phagocytosis
Flagellar assembly	219164	217059	Cellular Processes; Cell Motility; Flagellar assembly
Flavone and flavonol biosynthesis	98	83	Metabolism; Biosynthesis of Other Secondary Metabolites; Flavone and flavonol biosynthesis
Flavonoid biosynthesis	5489	4889	Metabolism; Biosynthesis of Other Secondary Metabolites; Flavonoid biosynthesis
Fluorobenzoate degradation	23521	23540	Metabolism; Xenobiotics Biodegradation and Metabolism; Fluorobenzoate degradation
Focal adhesion	3	0	Cellular Processes; Cell Communication; Focal adhesion

Folate biosynthesis	127668	126240	Metabolism; Metabolism of Cofactors and Vitamins; Folate biosynthesis
Fructose and mannose metabolism	191688	193358	Metabolism; Carbohydrate Metabolism; Fructose and mannose metabolism
Function unknown	554049	574876	Unclassified; Poorly Characterized; Function unknown
G protein-coupled receptors	41	38	Environmental Information Processing; Signaling Molecules and Interaction; G protein-coupled receptors
Galactose metabolism	127796	129493	Metabolism; Carbohydrate Metabolism; Galactose metabolism
General function prediction only	1051054	1030615	Unclassified; Poorly Characterized; General function prediction only
Geraniol degradation	122235	107841	Metabolism; Metabolism of Terpenoids and Polyketides; Geraniol degradation
Germination	7219	2834	Unclassified; Cellular Processes and Signaling; Germination
Glutamatergic synapse	25197	22076	Organismal Systems; Nervous System; Glutamatergic synapse
Glutathione metabolism	170412	173126	Metabolism; Metabolism of Other Amino Acids; Glutathione metabolism
Glycan biosynthesis and metabolism	22543	26326	Unclassified; Metabolism; Glycan biosynthesis and metabolism
Glycerolipid metabolism	100310	94541	Metabolism; Lipid Metabolism; Glycerolipid metabolism
Glycerophospholipid metabolism	153173	149735	Metabolism; Lipid Metabolism; Glycerophospholipid metabolism
Glycine, serine and threonine metabolism	282768	268050	Metabolism; Amino Acid Metabolism; Glycine, serine and threonine metabolism
Glycolysis / Gluconeogenesis	320875	309051	Metabolism; Carbohydrate Metabolism; Glycolysis / Gluconeogenesis
Glycosaminoglycan degradation	8853	9555	Metabolism; Glycan Biosynthesis and Metabolism; Glycosaminoglycan degradation
Glycosphingolipid biosynthesis - ganglio series	5659	5978	Metabolism; Glycan Biosynthesis and Metabolism; Glycosphingolipid biosynthesis - ganglio series
Glycosphingolipid biosynthesis - globo series	11336	11202	Metabolism; Glycan Biosynthesis and Metabolism; Glycosphingolipid biosynthesis - globo series
Glycosphingolipid biosynthesis - lacto and neolacto series	298	287	Metabolism; Glycan Biosynthesis and Metabolism; Glycosphingolipid biosynthesis - lacto and neolacto series
Glycosylphosphatidylinositol(GPI)-anchor biosynthesis	2	4	Metabolism; Glycan Biosynthesis and Metabolism; Glycosylphosphatidylinositol(GPI)-anchor biosynthesis
Glycosyltransferases	106532	108387	Metabolism; Glycan Biosynthesis and Metabolism; Glycosyltransferases
Glyoxylate and dicarboxylate metabolism	252603	244512	Metabolism; Carbohydrate Metabolism; Glyoxylate and dicarboxylate metabolism
GnRH signaling pathway	14	8	Organismal Systems; Endocrine System; GnRH signaling pathway
Hematopoietic cell lineage	19	21	Organismal Systems; Immune System; Hematopoietic cell lineage
Histidine metabolism	156549	145249	Metabolism; Amino Acid Metabolism; Histidine metabolism
Homologous recombination	187689	183896	Genetic Information Processing; Replication and Repair; Homologous recombination
Huntington's disease	41062	42226	Human Diseases; Neurodegenerative Diseases; Huntington's disease
Hypertrophic cardiomyopathy (HCM)	687	819	Human Diseases; Cardiovascular Diseases; Hypertrophic cardiomyopathy (HCM)
Indole alkaloid biosynthesis	227	219	Metabolism; Biosynthesis of Other Secondary Metabolites; Indole alkaloid biosynthesis
Influenza A	10856	10859	Human Diseases; Infectious Diseases; Influenza A
Inorganic ion transport and metabolism	121883	126264	Unclassified; Cellular Processes and Signaling; Inorganic ion transport and metabolism
Inositol phosphate metabolism	67963	68538	Metabolism; Carbohydrate Metabolism; Inositol phosphate metabolism
Insulin signaling pathway	15523	14766	Organismal Systems; Endocrine System; Insulin signaling pathway
Ion channels	8844	8143	Environmental Information Processing; Signaling Molecules and Interaction; Ion channels

Isoflavonoid biosynthesis	65	81	Metabolism; Biosynthesis of Other Secondary Metabolites; Isoflavonoid biosynthesis
Isoquinoline alkaloid biosynthesis	18771	17901	Metabolism; Biosynthesis of Other Secondary Metabolites; Isoquinoline alkaloid biosynthesis
Leishmaniasis	3	2	Human Diseases; Infectious Diseases; Leishmaniasis
Leukocyte transendothelial migration	2	0	Organismal Systems; Immune System; Leukocyte transendothelial migration
Limonene and pinene degradation	124806	106823	Metabolism; Metabolism of Terpenoids and Polyketides; Limonene and pinene degradation
Linoleic acid metabolism	17996	12900	Metabolism; Lipid Metabolism; Linoleic acid metabolism
Lipid biosynthesis proteins	224010	209515	Metabolism; Lipid Metabolism; Lipid biosynthesis proteins
Lipid metabolism	43006	46550	Unclassified; Metabolism; Lipid metabolism
Lipoic acid metabolism	19239	19067	Metabolism; Metabolism of Cofactors and Vitamins; Lipoic acid metabolism
Lipopolysaccharide biosynthesis	70168	81610	Metabolism; Glycan Biosynthesis and Metabolism; Lipopolysaccharide biosynthesis
Lipopolysaccharide biosynthesis proteins	108098	127979	Metabolism; Glycan Biosynthesis and Metabolism; Lipopolysaccharide biosynthesis proteins
Lysine biosynthesis	172254	167976	Metabolism; Amino Acid Metabolism; Lysine biosynthesis
Lysine degradation	167440	153704	Metabolism; Amino Acid Metabolism; Lysine degradation
Lysosome	14017	14966	Cellular Processes; Transport and Catabolism; Lysosome
MAPK signaling pathway - yeast	12732	12841	Environmental Information Processing; Signal Transduction; MAPK signaling pathway - yeast
Meiosis - yeast	14535	10199	Cellular Processes; Cell Growth and Death; Meiosis - yeast
Melanogenesis	78	58	Organismal Systems; Endocrine System; Melanogenesis
Membrane and intracellular structural molecules	232502	254904	Unclassified; Cellular Processes and Signaling; Membrane and intracellular structural molecules
Metabolism of cofactors and vitamins	65313	65408	Unclassified; Metabolism; Metabolism of cofactors and vitamins
Metabolism of xenobiotics by cytochrome P450	79804	79020	Metabolism; Xenobiotics Biodegradation and Metabolism; Metabolism of xenobiotics by cytochrome P450
Methane metabolism	285822	271282	Metabolism; Energy Metabolism; Methane metabolism
Mineral absorption	3442	2731	Organismal Systems; Digestive System; Mineral absorption
Mismatch repair	170389	163196	Genetic Information Processing; Replication and Repair; Mismatch repair
N-Glycan biosynthesis	8890	7378	Metabolism; Glycan Biosynthesis and Metabolism; N-Glycan biosynthesis
NOD-like receptor signaling pathway	4436	4311	Organismal Systems; Immune System; NOD-like receptor signaling pathway
Naphthalene degradation	79553	67666	Metabolism; Xenobiotics Biodegradation and Metabolism; Naphthalene degradation
Neuroactive ligand-receptor interaction	14	5	Environmental Information Processing; Signaling Molecules and Interaction; Neuroactive ligand-receptor interaction
Nicotinate and nicotinamide metabolism	127550	125848	Metabolism; Metabolism of Cofactors and Vitamins; Nicotinate and nicotinamide metabolism
Nitrogen metabolism	218601	217500	Metabolism; Energy Metabolism; Nitrogen metabolism
Nitrotoluene degradation	26554	21513	Metabolism; Xenobiotics Biodegradation and Metabolism; Nitrotoluene degradation
Non-homologous end-joining	19315	13170	Genetic Information Processing; Replication and Repair; Non-homologous end-joining
Notch signaling pathway	26	2	Environmental Information Processing; Signal Transduction; Notch signaling pathway
Novobiocin biosynthesis	36512	36099	Metabolism; Biosynthesis of Other Secondary Metabolites; Novobiocin biosynthesis
Nucleotide excision repair	87330	77345	Genetic Information Processing; Replication and Repair; Nucleotide excision repair
Nucleotide metabolism	17396	20962	Unclassified; Metabolism; Nucleotide metabolism

One carbon pool by folate	133790	130899	Metabolism; Metabolism of Cofactors and Vitamins; One carbon pool by folate
Other glycan degradation	29761	31313	Metabolism; Glycan Biosynthesis and Metabolism; Other glycan degradation
Other ion-coupled transporters	385748	405554	Unclassified; Cellular Processes and Signaling; Other ion-coupled transporters
Other transporters	74807	82256	Unclassified; Cellular Processes and Signaling; Other transporters
Others	325688	313254	Unclassified; Metabolism; Others
Oxidative phosphorylation	382286	375190	Metabolism; Energy Metabolism; Oxidative phosphorylation
PPAR signaling pathway	58706	50473	Organismal Systems; Endocrine System; PPAR signaling pathway
Pancreatic secretion	14	5	Organismal Systems; Digestive System; Pancreatic secretion
Pantothenate and CoA biosynthesis	157710	153263	Metabolism; Metabolism of Cofactors and Vitamins; Pantothenate and CoA biosynthesis
Parkinson's disease	24533	24486	Human Diseases; Neurodegenerative Diseases; Parkinson's disease
Pathogenic Escherichia coli infection	2	0	Human Diseases; Infectious Diseases; Pathogenic Escherichia coli infection
Pathways in cancer	23646	23282	Human Diseases; Cancers; Pathways in cancer
Penicillin and cephalosporin biosynthesis	16519	16531	Metabolism; Biosynthesis of Other Secondary Metabolites; Penicillin and cephalosporin biosynthesis
Pentose and glucuronate interconversions	151328	151407	Metabolism; Carbohydrate Metabolism; Pentose and glucuronate interconversions
Pentose phosphate pathway	222603	218915	Metabolism; Carbohydrate Metabolism; Pentose phosphate pathway
Peptidases	457471	454581	Metabolism; Enzyme Families; Peptidases
Peptidoglycan biosynthesis	172213	167379	Metabolism; Glycan Biosynthesis and Metabolism; Peptidoglycan biosynthesis
Peroxisome	80275	73873	Cellular Processes; Transport and Catabolism; Peroxisome
Pertussis	29245	38148	Human Diseases; Infectious Diseases; Pertussis
Phagosome	2	0	Cellular Processes; Transport and Catabolism; Phagosome
Phenylalanine metabolism	133372	125286	Metabolism; Amino Acid Metabolism; Phenylalanine metabolism
Phenylalanine, tyrosine and tryptophan biosynthesis	187297	187031	Metabolism; Amino Acid Metabolism; Phenylalanine, tyrosine and tryptophan biosynthesis
Phenylpropanoid biosynthesis	38482	40281	Metabolism; Biosynthesis of Other Secondary Metabolites; Phenylpropanoid biosynthesis
Phosphatidylinositol signaling system	28614	26390	Environmental Information Processing; Signal Transduction; Phosphatidylinositol signaling system
Phosphonate and phosphinate metabolism	20085	20786	Metabolism; Metabolism of Other Amino Acids; Phosphonate and phosphinate metabolism
Phosphotransferase system (PTS)	130735	159632	Environmental Information Processing; Membrane Transport; Phosphotransferase system (PTS)
Photosynthesis	74593	72329	Metabolism; Energy Metabolism; Photosynthesis
Photosynthesis - antenna proteins	102	72	Metabolism; Energy Metabolism; Photosynthesis - antenna proteins
Photosynthesis proteins	83116	80822	Metabolism; Energy Metabolism; Photosynthesis proteins
Phototransduction - fly	2	0	Organismal Systems; Sensory System; Phototransduction - fly
Plant-pathogen interaction	36924	37233	Organismal Systems; Environmental Adaptation; Plant-pathogen interaction
Polycyclic aromatic hydrocarbon degradation	41852	32155	Metabolism; Xenobiotics Biodegradation and Metabolism; Polycyclic aromatic hydrocarbon degradation
Polyketide sugar unit biosynthesis	34787	34407	Metabolism; Metabolism of Terpenoids and Polyketides; Polyketide sugar unit biosynthesis
Pores ion channels	196035	221591	Unclassified; Cellular Processes and Signaling; Pores ion channels
Porphyrin and chlorophyll metabolism	266305	261010	Metabolism; Metabolism of Cofactors and Vitamins; Porphyrin and chlorophyll metabolism

Prenyltransferases	81839	75752	Metabolism; Metabolism of Terpenoids and Polyketides; Prenyltransferases
Primary bile acid biosynthesis	4038	2973	Metabolism; Lipid Metabolism; Primary bile acid biosynthesis
Primary immunodeficiency	13785	12457	Human Diseases; Immune System Diseases; Primary immunodeficiency
Prion diseases	5090	5782	Human Diseases; Neurodegenerative Diseases; Prion diseases
Progesterone-mediated oocyte maturation	4409	4298	Organismal Systems; Endocrine System; Progesterone-mediated oocyte maturation
Propanoate metabolism	306168	284655	Metabolism; Carbohydrate Metabolism; Propanoate metabolism
Prostate cancer	4413	4303	Human Diseases; Cancers; Prostate cancer
Proteasome	7137	5559	Genetic Information Processing; Folding, Sorting and Degradation; Proteasome
Protein digestion and absorption	2769	3023	Organismal Systems; Digestive System; Protein digestion and absorption
Protein export	143340	137261	Genetic Information Processing; Folding, Sorting and Degradation; Protein export
Protein folding and associated processing	223372	228419	Unclassified; Genetic Information Processing; Protein folding and associated processing
Protein kinases	124996	125669	Metabolism; Enzyme Families; Protein kinases
Protein processing in endoplasmic reticulum	12701	11607	Genetic Information Processing; Folding, Sorting and Degradation; Protein processing in endoplasmic reticulum
Proximal tubule bicarbonate reclamation	8966	8736	Organismal Systems; Excretory System; Proximal tubule bicarbonate reclamation
Purine metabolism	583293	563071	Metabolism; Nucleotide Metabolism; Purine metabolism
Pyrimidine metabolism	379689	366934	Metabolism; Nucleotide Metabolism; Pyrimidine metabolism
Pyruvate metabolism	340996	333323	Metabolism; Carbohydrate Metabolism; Pyruvate metabolism
RIG-I-like receptor signaling pathway	2549	1662	Organismal Systems; Immune System; RIG-I-like receptor signaling pathway
RNA degradation	115706	113808	Genetic Information Processing; Folding, Sorting and Degradation; RNA degradation
RNA polymerase	34529	32601	Genetic Information Processing; Transcription; RNA polymerase
RNA transport	14629	16221	Genetic Information Processing; Translation; RNA transport
Regulation of actin cytoskeleton	2	0	Cellular Processes; Cell Motility; Regulation of actin cytoskeleton
Renal cell carcinoma	8368	8126	Human Diseases; Cancers; Renal cell carcinoma
Renin-angiotensin system	1654	1333	Organismal Systems; Endocrine System; Renin-angiotensin system
Replication, recombination and repair proteins	213765	211362	Unclassified; Genetic Information Processing; Replication, recombination and repair proteins
Restriction enzyme	19261	21409	Unclassified; Genetic Information Processing; Restriction enzyme
Retinol metabolism	30498	28762	Metabolism; Metabolism of Cofactors and Vitamins; Retinol metabolism
Riboflavin metabolism	84451	83947	Metabolism; Metabolism of Cofactors and Vitamins; Riboflavin metabolism
Ribosome	458258	438646	Genetic Information Processing; Translation; Ribosome
Ribosome Biogenesis	318931	324262	Genetic Information Processing; Translation; Ribosome Biogenesis
Ribosome biogenesis in eukaryotes	12719	12123	Genetic Information Processing; Translation; Ribosome biogenesis in eukaryotes
Secondary bile acid biosynthesis	1154	883	Metabolism; Lipid Metabolism; Secondary bile acid biosynthesis
Secretion system	594481	638678	Environmental Information Processing; Membrane Transport; Secretion system
Selenocompound metabolism	107656	106624	Metabolism; Metabolism of Other Amino Acids; Selenocompound metabolism
Sesquiterpenoid biosynthesis	31	10	Metabolism; Metabolism of Terpenoids and Polyketides; Sesquiterpenoid biosynthesis
Shigellosis	101	254	Human Diseases; Infectious Diseases; Shigellosis
Signal transduction mechanisms	138597	142904	Unclassified; Cellular Processes and Signaling; Signal transduction mechanisms
Small cell lung cancer	10843	10856	Human Diseases; Cancers; Small cell lung cancer

Sphingolipid metabolism	20440	20018	Metabolism; Lipid Metabolism; Sphingolipid metabolism
Sporulation	34209	19078	Unclassified; Cellular Processes and Signaling; Sporulation
Staphylococcus aureus infection	4572	3897	Human Diseases; Infectious Diseases; Staphylococcus aureus infection
Starch and sucrose metabolism	210705	214844	Metabolism; Carbohydrate Metabolism; Starch and sucrose metabolism
Steroid biosynthesis	2459	2081	Metabolism; Lipid Metabolism; Steroid biosynthesis
Steroid hormone biosynthesis	8642	7933	Metabolism; Lipid Metabolism; Steroid hormone biosynthesis
Stilbenoid, diarylheptanoid and gingerol biosynthesis	9260	6099	Metabolism; Biosynthesis of Other Secondary Metabolites; Stilbenoid, diarylheptanoid and gingerol biosynthesis
Streptomycin biosynthesis	77150	74395	Metabolism; Biosynthesis of Other Secondary Metabolites; Streptomycin biosynthesis
Styrene degradation	39400	36520	Metabolism; Xenobiotics Biodegradation and Metabolism; Styrene degradation
Sulfur metabolism	102251	101777	Metabolism; Energy Metabolism; Sulfur metabolism
Sulfur relay system	98310	98389	Genetic Information Processing; Folding, Sorting and Degradation; Sulfur relay system
Synthesis and degradation of ketone bodies	56010	52430	Metabolism; Lipid Metabolism; Synthesis and degradation of ketone bodies
Systemic lupus erythematosus	151	161	Human Diseases; Immune System Diseases; Systemic lupus erythematosus
Taurine and hypotaurine metabolism	41194	41235	Metabolism; Metabolism of Other Amino Acids; Taurine and hypotaurine metabolism
Terpenoid backbone biosynthesis	138051	129287	Metabolism; Metabolism of Terpenoids and Polyketides; Terpenoid backbone biosynthesis
Tetracycline biosynthesis	37137	36900	Metabolism; Metabolism of Terpenoids and Polyketides; Tetracycline biosynthesis
Thiamine metabolism	103728	101789	Metabolism; Metabolism of Cofactors and Vitamins; Thiamine metabolism
Tight junction	2	0	Cellular Processes; Cell Communication; Tight junction
Toluene degradation	68588	67153	Metabolism; Xenobiotics Biodegradation and Metabolism; Toluene degradation
Toxoplasmosis	10840	10869	Human Diseases; Infectious Diseases; Toxoplasmosis
Transcription factors	528758	551448	Genetic Information Processing; Transcription; Transcription factors
Transcription machinery	202274	192314	Genetic Information Processing; Transcription; Transcription machinery
Transcription related proteins	4108	5329	Unclassified; Genetic Information Processing; Transcription related proteins
Translation factors	104440	103338	Genetic Information Processing; Translation; Translation factors
Translation proteins	203172	209171	Unclassified; Genetic Information Processing; Translation proteins
Transporters	1956336	1780833	Environmental Information Processing; Membrane Transport; Transporters
Tropane, piperidine and pyridine alkaloid biosynthesis	37828	35249	Metabolism; Biosynthesis of Other Secondary Metabolites; Tropane, piperidine and pyridine alkaloid biosynthesis
Tryptophan metabolism	211750	193192	Metabolism; Amino Acid Metabolism; Tryptophan metabolism
Tuberculosis	40780	38593	Human Diseases; Infectious Diseases; Tuberculosis
Two-component system	710706	735064	Environmental Information Processing; Signal Transduction; Two-component system
Type I diabetes mellitus	11448	9915	Human Diseases; Metabolic Diseases; Type I diabetes mellitus
Type II diabetes mellitus	10691	10881	Human Diseases; Metabolic Diseases; Type II diabetes mellitus
Tyrosine metabolism	151929	142109	Metabolism; Amino Acid Metabolism; Tyrosine metabolism
Ubiquinone and other terpenoid-quinone biosynthesis	104409	108581	Metabolism; Metabolism of Cofactors and Vitamins; Ubiquinone and other terpenoid-quinone biosynthesis
Ubiquitin system	4068	3997	Genetic Information Processing; Folding, Sorting and Degradation; Ubiquitin system
VEGF signaling pathway	3	2	Environmental Information Processing; Signal Transduction; VEGF signaling pathway

Valine, leucine and isoleucine biosynthesis	204742	198500	Metabolism; Amino Acid Metabolism; Valine, leucine and isoleucine biosynthesis
Valine, leucine and isoleucine degradation	304808	277458	Metabolism; Amino Acid Metabolism; Valine, leucine and isoleucine degradation
Various types of N-glycan biosynthesis	2	1	Metabolism; Glycan Biosynthesis and Metabolism; Various types of N-glycan biosynthesis
Vasopressin-regulated water reabsorption	4	2	Organismal Systems; Excretory System; Vasopressin-regulated water reabsorption
Vibrio cholerae infection	322	392	Human Diseases; Infectious Diseases; Vibrio cholerae infection
Vibrio cholerae pathogenic cycle	27060	28594	Human Diseases; Infectious Diseases; Vibrio cholerae pathogenic cycle
Viral myocarditis	10842	10854	Human Diseases; Cardiovascular Diseases; Viral myocarditis
Vitamin B6 metabolism	55777	55272	Metabolism; Metabolism of Cofactors and Vitamins; Vitamin B6 metabolism
Wnt signaling pathway	26	2	Environmental Information Processing; Signal Transduction; Wnt signaling pathway
Xylene degradation	22660	23961	Metabolism; Xenobiotics Biodegradation and Metabolism; Xylene degradation
Zeatin biosynthesis	8590	8209	Metabolism; Metabolism of Terpenoids and Polyketides; Zeatin biosynthesis
alpha-Linolenic acid metabolism	14389	14132	Metabolism; Lipid Metabolism; alpha-Linolenic acid metabolism
beta-Alanine metabolism	159674	145472	Metabolism; Metabolism of Other Amino Acids; beta-Alanine metabolism
beta-Lactam resistance	11655	12501	Metabolism; Biosynthesis of Other Secondary Metabolites; beta-Lactam resistance
mRNA surveillance pathway	15	1	Genetic Information Processing; Translation; mRNA surveillance pathway
p53 signaling pathway	10874	10891	Cellular Processes; Cell Growth and Death; p53 signaling pathway
